# Broadly-Reactive Neutralizing and Non-neutralizing Antibodies Directed against the H7 Influenza Virus Hemagglutinin Reveal Divergent Mechanisms of Protection

**DOI:** 10.1371/journal.ppat.1005578

**Published:** 2016-04-15

**Authors:** Gene S. Tan, Paul E. Leon, Randy A. Albrecht, Irina Margine, Ariana Hirsh, Justin Bahl, Florian Krammer

**Affiliations:** 1 Department of Microbiology, Icahn School of Medicine at Mount Sinai, New York, New York, United States of America; 2 Graduate School of Biomedical Sciences, Icahn School of Medicine at Mount Sinai, New York, New York, United States of America; 3 Global Health and Emerging Pathogens Institute, Icahn School of Medicine at Mount Sinai, New York, New York, United States of America; 4 School of Public Health, The University of Texas Health Science Center at Houston, Houston, Texas, United States of America; St. Jude Children's Research Hospital, UNITED STATES

## Abstract

In the early spring of 2013, Chinese health authorities reported several cases of H7N9 influenza virus infections in humans. Since then the virus has established itself at the human-animal interface in Eastern China and continues to cause several hundred infections annually. In order to characterize the antibody response to the H7N9 virus we generated several mouse monoclonal antibodies against the hemagglutinin of the A/Shanghai/1/13 (H7N9) virus. Of particular note are two monoclonal antibodies, 1B2 and 1H5, that show broad reactivity to divergent H7 hemagglutinins. Monoclonal antibody 1B2 binds to viruses of the Eurasian and North American H7 lineages and monoclonal antibody 1H5 reacts broadly to virus isolates of the Eurasian lineage. Interestingly, 1B2 shows broad hemagglutination inhibiting and neutralizing activity, while 1H5 fails to inhibit hemagglutination and demonstrates no neutralizing activity *in vitro*. However, both monoclonal antibodies were highly protective in an *in vivo* passive transfer challenge model in mice, even at low doses. Experiments using mutant antibodies that lack the ability for Fc/Fc-receptor and Fc/complement interactions suggest that the protection provided by mAb 1H5 is, at least in part, mediated by the Fc-fragment of the mAb. These findings highlight that a protective response to a pathogen may not only be due to neutralizing antibodies, but can also be the result of highly efficacious non-neutralizing antibodies not readily detected by classical *in vitro* neutralization or hemagglutination inhibition assays. This is of interest because H7 influenza virus vaccines induce only low hemagglutination inhibiting antibody titers while eliciting robust antibody titers as measured by ELISA. Our data suggest that these binding but non-neutralizing antibodies contribute to protection *in vivo*.

## Author Summary

Several hundred human avian H7N9 virus infections with a case fatality rate of approximately 37% have occurred in China since 2013. The emergence of this virus has raised concerns about its pandemic potential and has triggered the development of H7N9 vaccines. Using the traditional correlate of protection for influenza viruses—the hemagglutination inhibition assay—H7N9 vaccines prove to be poorly immunogenic. However, once antibody levels are high enough substantial crossreactivity is observed. Here we characterize several monoclonal antibodies against the H7N9 hemagglutinin. One set of antibodies is active in the hemagglutination inhibition assay across a panel of distant H7 virus strains and the common conserved epitope of these antibodies might explain the broad crossreactivity observed with H7N9 vaccines. The second set of antibodies shows no antiviral activity *in vitro* but is highly protective *in vivo*, partially through Fc-mediated functions. Targeting of the non-neutralizing but protective epitope of these antibodies by the immune system might explain the 'low immunogenicity' observed in H7N9 vaccine trials. In conclusion our data suggests that current H7N9 vaccines could protect against divergent H7 strains with pandemic potential in the future and that the traditional correlates of protection for influenza vaccines might not capture the protective immunity to H7 viruses.

## Introduction

In 2013, zoonotic infections with H7N9 influenza virus were reported in Eastern China [1]. Since then numerous human cases have been confirmed, revealing a trend of local outbreaks coinciding with annual seasonal influenza virus epidemics [2]. Although human-to-human transmission of the virus has been limited so far [3–7], the reported case fatality for H7N9 infections appears high, specifically among older individuals. In addition, several co-infections of H7N9 with seasonal influenza A viruses have been reported raising concerns that a novel pandemic virus could emerge through re-assortment [8–10]. Therefore, several pre-pandemic H7N9 influenza virus vaccines have been developed and tested [11–13]. While H7 vaccines were shown to be immunogenic and to induce strong antibody responses as measured by enzyme linked immunosorbent assays (ELISA) [14, 15], the hemagglutination inhibition (HI) titers elicited (the major correlate of protection) were surprisingly low even when strong adjuvants were used [11–13, 16–18]. A second observation from H7 vaccine trials was that HI active antibodies appeared to be highly cross-reactive, even between very divergent isolates belonging to the Eurasian or North American H7 lineage [14, 19–22]. Thus far, it remains unknown which epitopes are targeted by cross-reactive antibodies detected by HI and ELISA. Furthermore, it is unclear whether antibodies detected by ELISA confer protection in the absence of high HI titers. In this study we characterize the breadth and mechanism of protection of a set of monoclonal antibodies (mAbs) raised against the H7 hemagglutinin (HA). The characterization of these monoclonal antibodies could help define the mechanism of action of the antibody responses observed following vaccination in humans.

## Results

### Antibody characterization: Binding and neutralization profiles

**Fig 1:**
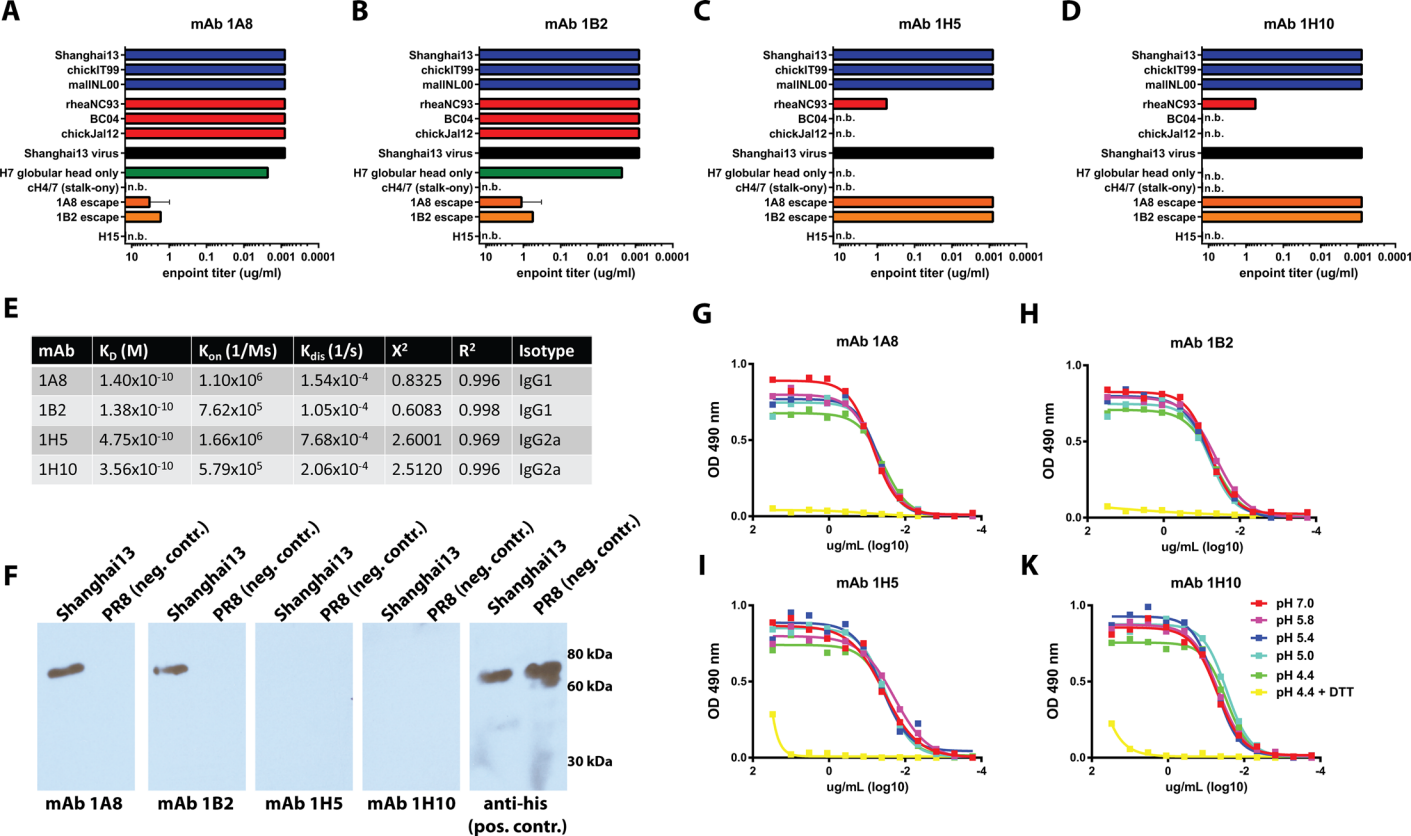
Characterization of mAbs 1A2, 1B8, 1H5 and 1H10. **A-D** Binding characteristics of the four mAbs to divergent recombinant HA and virus substrates (as indicated on the y-axis). Results are blotted as minimal binding concentrations measured by a quantitative ELISA method. n.b. no binding. **E** Binding kinetics of the four mAbs as measured using an Octet Red system and recombinant H7 Shanghai13 HA as substrate. **F** Western blot of Shangahi13 (H7) and PR8 (H1) HA proteins probed with mAbs 1B2, A18, 1H5 and 1H10 or an antibody against the hexahistidine-tag which is present on both recombinant proteins (positive control). **G-K** Binding of the four mAbs to Shanghai 13 virus that was pre-treated with buffers of different pH (as indicated in the legend) or a reducing agent (DTT).

We generated four monoclonal antibodies from an H7N9 immunized mouse through hybridoma fusion that reacted positively with recombinant Shanghai13 HA during the screening process (see Materials and Methods). These mAbs were purified and initially characterized for their breadth of binding. Two different binding patterns were observed: i) two of the mAbs 1A8 and 1B2 (both IgG_1_) showed strong binding to all tested recombinant H7 HAs, which included both European lineage isolates (Shanghai13, chickIT99 and mallNL09) as well as North American isolates (rheaNC93, BC04 and chickJal12) (Fig 1A and 1B); while the other two ii) mAbs 1H5 and 1H10 (both IgG_2a_) bound strongly to all Eurasian lineage HAs, but only weakly to one North American lineage isolate (rheaNC93 and not to the others) (Fig 1C and 1D). Interestingly, the monoclonal antibody pairs (1A8/1B2 and 1H5/1H10) are clonally related (having the same germ-line genes) but not identical (different somatic mutations in their variable regions). Of note, all mAbs generated also bound to the most recent Chinese H7 isolate, Shanghai13, but did not bind to an H15 HA. The broadly binding mAbs, 1A8 and 1B2, seem to target the globular head domain as demonstrated by their ability to bind to an H7 globular head (only) protein and not to the chimeric HA protein, cH4/7 (H4 globular head with and H7 stalk). On the other hand, mAbs 1H5 and 1H10 did not bind to either the H7 globular head construct or the chimeric HA, cH4/7. We also assessed the four mAbs using bio-layer interferometry and found sub-nanomolar KD values (Fig 1E and S1 Fig).

Next, we asked the question whether the epitopes recognized by these mAbs were linear or conformational. A Western blot assay using the Shanghai13 HA showed that mAbs 1A8 and 1B2 bound well under denaturing and reducing conditions, while mAbs 1H5 and 1H10 completely lost their binding (Fig 1F). To investigate this further, we wanted to assess if the epitopes of any of these mAbs would be denatured during the HA fusion process at low pH, which is characterized by major structural re-arrangements. However, all four mAbs bound well to both the pre- and post-fusion conformations of the H7 HA. Since binding of anti-stalk antibodies with conformational epitopes is ablated by pH treatment this finding suggests that mAbs 1H5 and 1H10 bind to the head domain [23, 24]. Subsequent treatment with the reducing agent DTT at low pH removed the HA1 subunit from the virion and ablated binding of all four mAbs (Fig 1G–1J).

**Fig 2:**
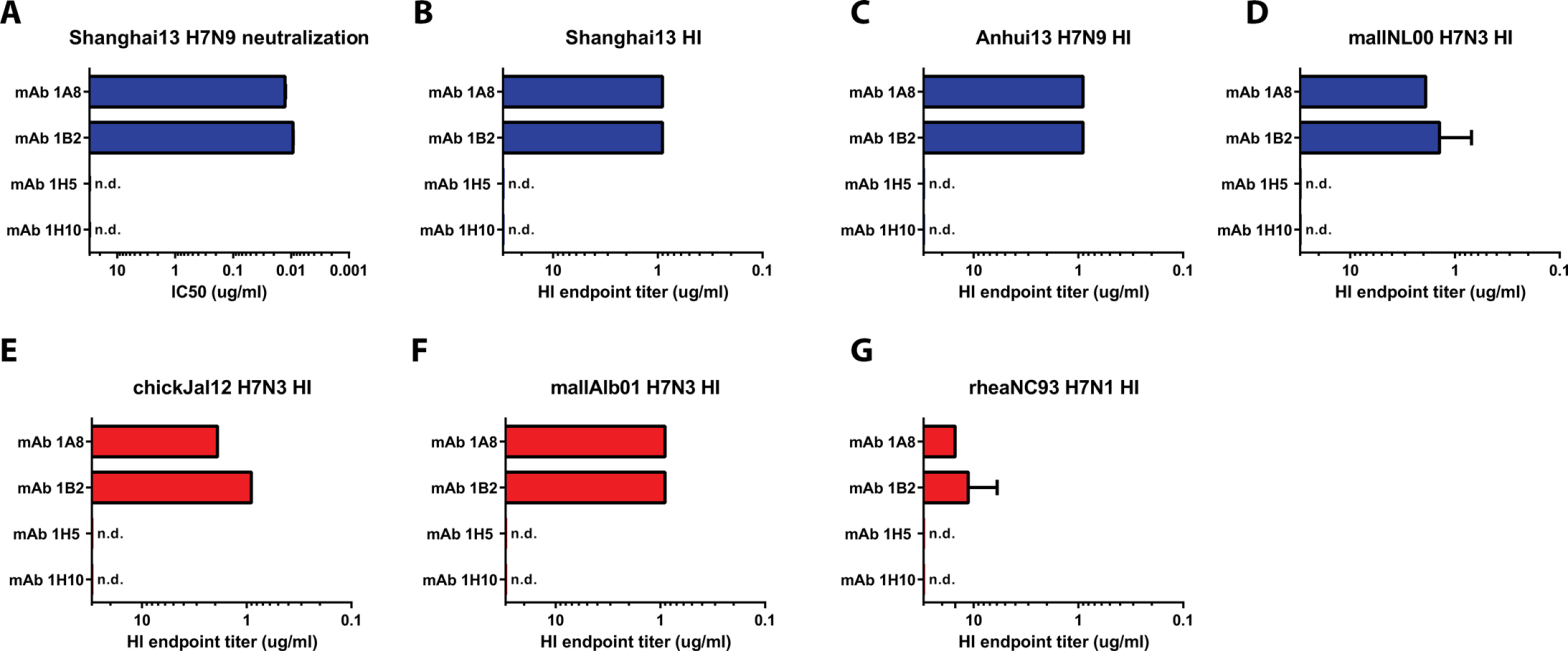
*In vitro* activity of mAbs 1B2, 1A8, 1H5 and 1H10. **A** Neutralization activity of mAbs 1B2, A18, 1H5 and 1H10 against Shanghai13 virus. Hemagglutination inhibition (HI) activity of the four mAbs is shown in **B-G**. Blue bars indicate Eurasian lineage strains, red bars indicate North American lineage strains. n.d. not detected.

To characterize the functionality of the mAbs, we assessed their ability to interfere with receptor binding of the HA in an HI assay. MAbs 1A8 and 1B2 showed strong HI activity against all tested viruses including Shanghai13, Anhui13 and mallNL00 (Eurasian lineage) and chickJal12, mallAlb01 and rheaNC93 (North American lineage). HI activity was strong against five of the six viruses (endpoint concentrations of 1–3 ug/ml), but weaker against rheaNC93 virus (10–12 ug/ml) (Fig 2). MAbs 1H5 and 1H10 did not show HI activity against any of the tested viruses (Fig 2).

Finally, we assessed whether any of the mAbs had neutralizing activity *in vitro*. The mAbs were tested in a classical MN assay at a starting concentration of 300 ug/ml. Importantly, the antibodies in this assay remained in the overlay at all times during the incubation period to catch possible antiviral effects downstream of entry. MAbs 1A8 and 1B2 showed–as expected—strong neutralizing activities with IC_50_ values of 12.8 ng/ml and 9.2 ng/ml, respectively (Fig 2 and S2 Fig). However, mAbs 1H5 and 1H10 exhibited negligible neutralizing activity and never reached a 50% inhibition endpoint (Fig 2 and S2 Fig).

### Protective efficacy *in vivo*

**Fig 3:**
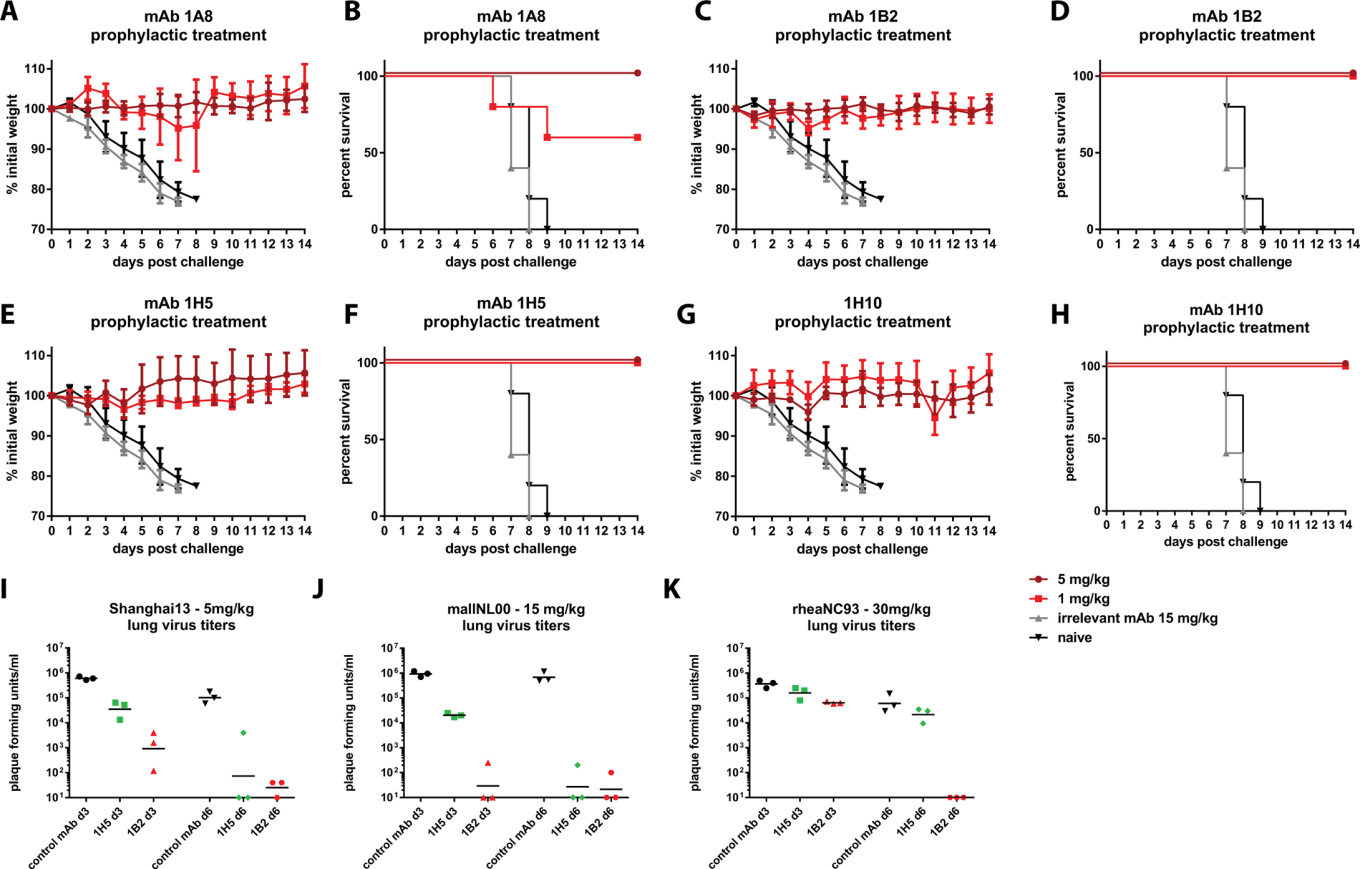
Prophylactic efficacy in the mouse model. Animals were pre-treated with 5 or 1 mg/kg of mAb and then challenged with Shanghai13 virus. **A, C, E and G** shows weight loss curves for the four mAbs, survival is shown in panels **B, D, F and H**. Negative control groups were the same for all experiments shown in **A-H**. **I-K** since the experiments were done simultaneously shows lung virus titers on day 3 and day 6 for mAbs 1B2 and 1H5 after challenge with Shanghai13 (**I**), mallNL00 (**J**) and rheaNC99 viruses(**K**). 5 mice per group were used for experiments shown in A-H, 3 mice per group were used for experiments shown in I-K.

To test if the four mAbs have protective activity *in vivo*, we performed prophylactic and therapeutic passive transfer challenge experiments in the mouse model. First we performed a Shanghai13 challenge experiment with mice prophylactically receiving 1 or 5 mg/kg of the respective mAbs. MAb 1A8 was completely protective at 5 mg/kg, but mice showed signs of disease (weight loss) in the 1 mg/kg group and survival was only 60%—despite the high *in vitro* neutralization activity of mAb 1A8 (Fig 3A and 3B). MAb 1B2 had better protective activity and prevented morbidity and mortality at both doses (Fig 3C and 3D). Remarkably, the non-neutralizing mAbs 1H5 and 1H10 also showed full protection against morbidity and mortality at both tested treatment doses (Fig 3E–3H). A third H7 HA binding non-neutralizing antibody of the same isotype as 1H5 and 1H10 did not protect from viral challenge (S3 Fig).

To investigate reduction in lung virus titer, we focused on mAbs 1B2 and 1H5 and performed another passive transfer/challenge experiment. Mice were treated with 5 mg/kg of the respective antibody, challenged and lungs were harvested on day 3 and day 5 post infection. Neutralizing mAb 1B2 reduced lung titers approximately 660-fold as compared to control animals that were treated with an irrelevant antibody as control (Fig 3I). Although virus was still detectable in 2 out of 3 mice on day 6 post infection, the titers were barely above the limit of detection and again more than 3 logs lower than in the controls. MAb 1H5 reduced lung titers on day 3 about 17-fold and virus was undetectable in 2 out of 3 animals on day 6 post infection—a remarkable finding for a non-neutralizing mAb (Fig 3I).

In order to assess the protective breadth of mAbs 1B2 and 1H5 we also performed passive transfer challenge experiments with mallNL00 (Eurasian lineage) and rheaNC93 (North American lineage) viruses. Since both of these strains exhibited low pathogenicity in mice we chose to use virus titers in lung homogenates as a readout. For mallNL00 mAb 1B2 reduced the viral load 5 logs on day 3 post infection with virus detectable at a low level in only one of three mice (Fig 3J). Similar results were obtained on day 6 post infection. MAb 1H5 reduced mouse lung titers by almost 2 logs on day 3 post infection and virus was not detectable in two out of three mice on day 6 post infection (Fig 3J). These results are similar to the data from the Shanghai13 challenge experiment. MAb 1B2 reduced the lung titers of rheaNC93 on day 3 post infection about 7-fold and eliminated virus entirely on day 6 post infection (Fig 3K). MAb 1H5 had—as expected from the binding data—no effect on the replication of rheaNC93 virus *in vivo* (Fig 3K).

**Fig 4:**
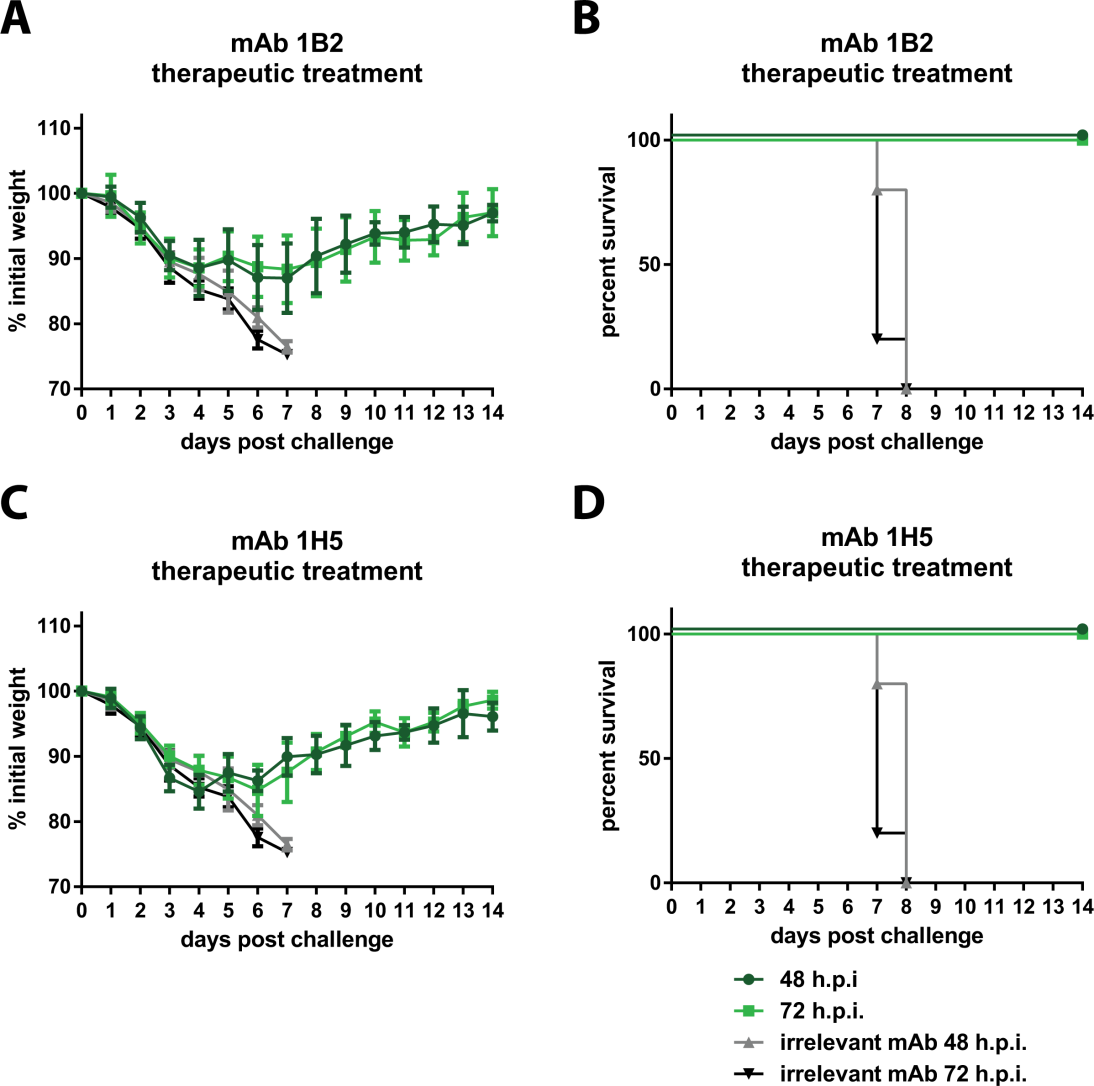
Therapeutic efficacy in the mouse model. Animals were infected with Shanghai13 virus and then treated therapeutically 48 or 72 hours post infection with mAbs 1B2 or 1H5 respectively. **A and C** show weight loss upon challenge, **B** and **D** show survival. The negative control groups are the same for both mAbs since the experiments were done simultaneously. 5 mice per group were used for experiments shown in this figure.

Finally, we characterized mAbs 1B2 and 1H5 in a therapeutic setting and assessed whether differences between neutralizing and non-neutralizing mAbs would be observed. Mice were infected with Shanghai13 virus and then treated with the respective mAbs 48 or 72 hours post infection. In both cases, 1B2- and 1H5- administered mice lost 10–15% of their initial body weight, and started to regain weight between day 5 and day 7 post infection (Fig 4A and 4C). Thus, in a therapeutic regimen, treatment with either mAb 1B2 (neutralizing) or mAb 1H5 (non-neutralizing) at 48 or 72 hours post infection completely protected mice from mortality (Fig 4B and 4D).

### Epitope analysis and characterization of escape mutants

**Fig 5:**
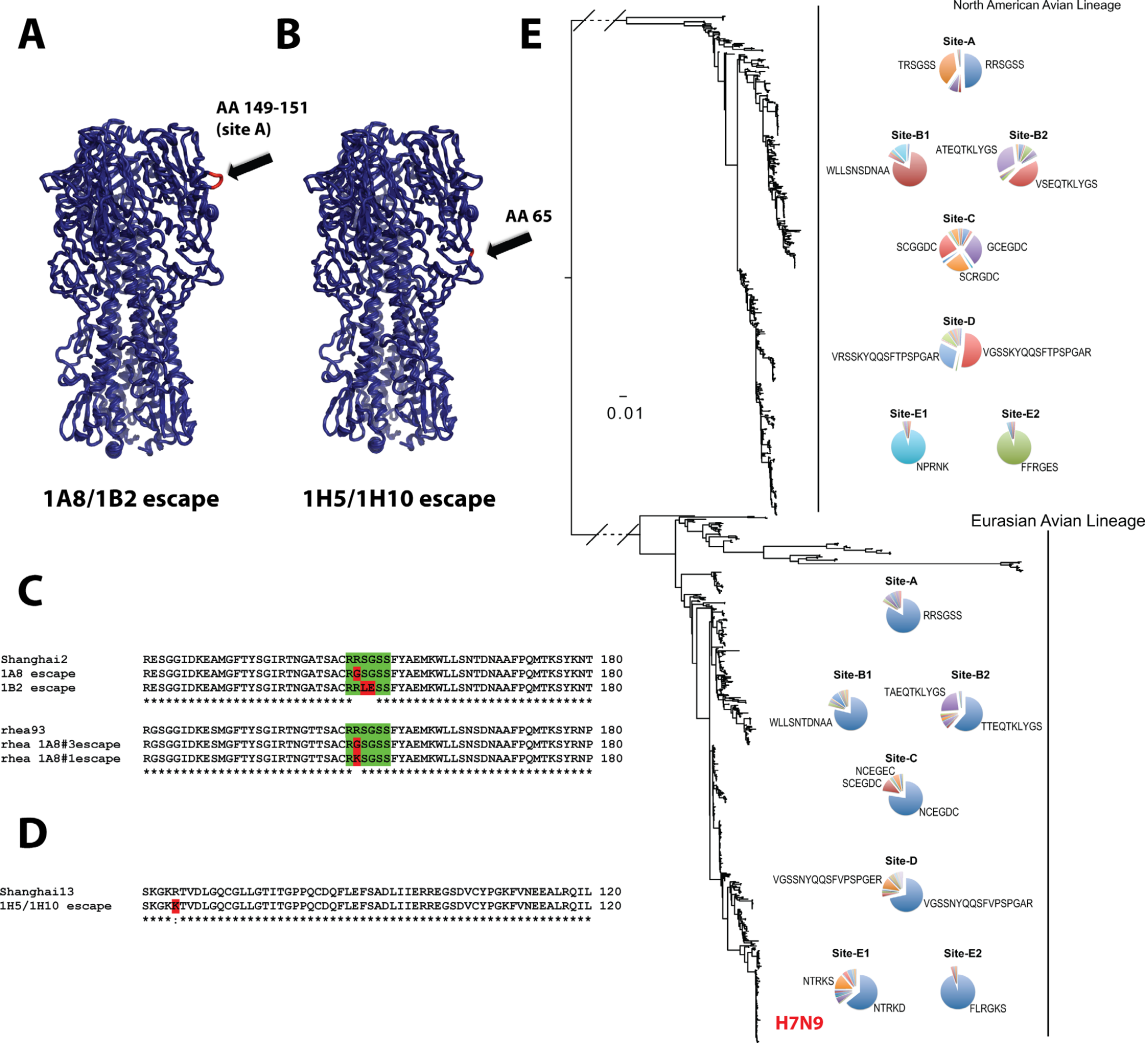
Epitope mapping and conservation. Panels **A** shows a graphic representation of the crystal structure of Shanghai13 HA (adapted from [6], PDB 4LCX) with the region that carries mAb 1A8/1B2 escape mutations (antigenic site A) indicated in red and by an arrow. **B** shows the same representation but with the 1H5 escape mutation indicated in red and by an arrow. **C and D** show sequence alignments of the 1A8/1B2 escape mutations (**C**) and of the 1H5 escape mutations (**D**). Mutations are indicated in red. Panel **E** shows a phylogenetic tree based on the amino acid sequence of the putative antigenic sites of H7 HA. A clear split between North American and Eurasian lineage is visible. Percentages of each variant for each of the antigenic sites are represented by pie charts with sequences annotated for the larger fractions. The position of H7N9 is indicated on the Eurasian lineage branch.

Since mAbs 1A8 and 1B2 exhibit neutralizing activity it was possible to generate *in vitro* escape mutants by incubating a large number of virions with neutralizing concentrations of mAbs and then injecting the mixture into embryonated eggs selecting for neutralization resistant variants. We obtained sequences from several plaque purified escape mutants of the Shanghai2 and rheaNC93 strains. Escape mutations following selection with either the mAb 1A8 or mAb 1B2 were localized in a site homologous to the antigenic site A of H3 HAs. MAb 1A8 selected for an R149G mutation (all H7 numbering starting from the first methionine) in the Shanghai2 HA and R149G or R149K in the rheaNC93 HA, respectively. MAb 1B2 selected for two simultaneous mutations in the Shanghai2 HA, S150L/G151E (Fig 5). Binding of both 1A8 and 1B2 to HA proteins that carried the R149G or S150L/G151E escape mutations was significantly diminished (Fig 1A and 1B).

Comparative genetic analysis of all available H7 HA sequences revealed, that the putative antigenic site A was highly conserved in both the American and the Eurasian H7 lineage (Fig 5E). The 1A8 and 1B2 mAbs bound to RRSGSS, which was found in 83.3% of all Eurasian lineage H7 HAs and in 49.4% of the North American isolates (S1 Table and S2 Table). This motif was the most common among all antigenic site A variants and was the dominant variant shared between the Eurasian and North American lineages. This suggests that avian immune systems either did not target this site or that this site has been functionally conserved and subjected to strong purifying selection. All other putative antigenic sites were highly divergent between lineages. We observed 72–99% (S3 Table) frequency of the RRSGSS antigenic site A variant among recent isolates per year.

Since mAbs 1H5 and 1H10 did not exhibit HI activity, we speculated that these mAbs would bind at some distance from the receptor binding site of the HA. Both mAbs did not bind to the H7 head-only construct but bound to the post-fusion HA. This data suggests that the (initial) formation of the epitope of non-neutralizing mAbs 1H5 and 1H10 may require that both the globular head and the stalk region are intact while denaturation of the HA trimer by acid treatment (to induce post-fusion conformation) does not impact the correct folding of the epitope An example for this phenomenon has recently been described for H1 binding antibodies [25]. Also, 1H5 and 1H10 did not show any diminished binding to 1A8- or 1B2-generated escape mutants suggesting that they did not bind to antigenic site A. Finally, we used a panel of H7 HA mutants that have been previously generated in our laboratory and screened them for decreased binding of mAb 1H5 and 1H10. We found diminished binding (S4 Fig and S5 Fig) against a mutant that carried the R65K mutation. This site is located at the lateral part of the global head domain in close proximity to the stalk domain and this finding suggests that mAb 1H5 and 1H10 are binding at the interface between the HA head and stalk domain. Furthermore, we wanted to test if 1H5/1H10-like antibodies are present in sera of H7 vaccinated human individuals. A competition ELISA revealed that both 1H5 and 1H10 competed strongly (between 17% and 27%) with human reactivity to H7 protein (S6 Fig).

Finally, we were interested to assess the impact of viral fitness by escape to the neutralizing and non-neutralizing antibodies. We tested the pathogenicity of the 1B2, 1A8 and R65K escape mutants in comparison to the parental viruses. Interestingly, all three escape mutants showed an attenuated phenotype which was strongest for the 1A8 escape virus which did not induce any weight loss/morbidity in mice even at the highest dose tested (10^5^ PFU) (S4 Table).

### The protective effect of mAb 1H5 is partially dependent on its Fc-fragment

**Fig 6:**
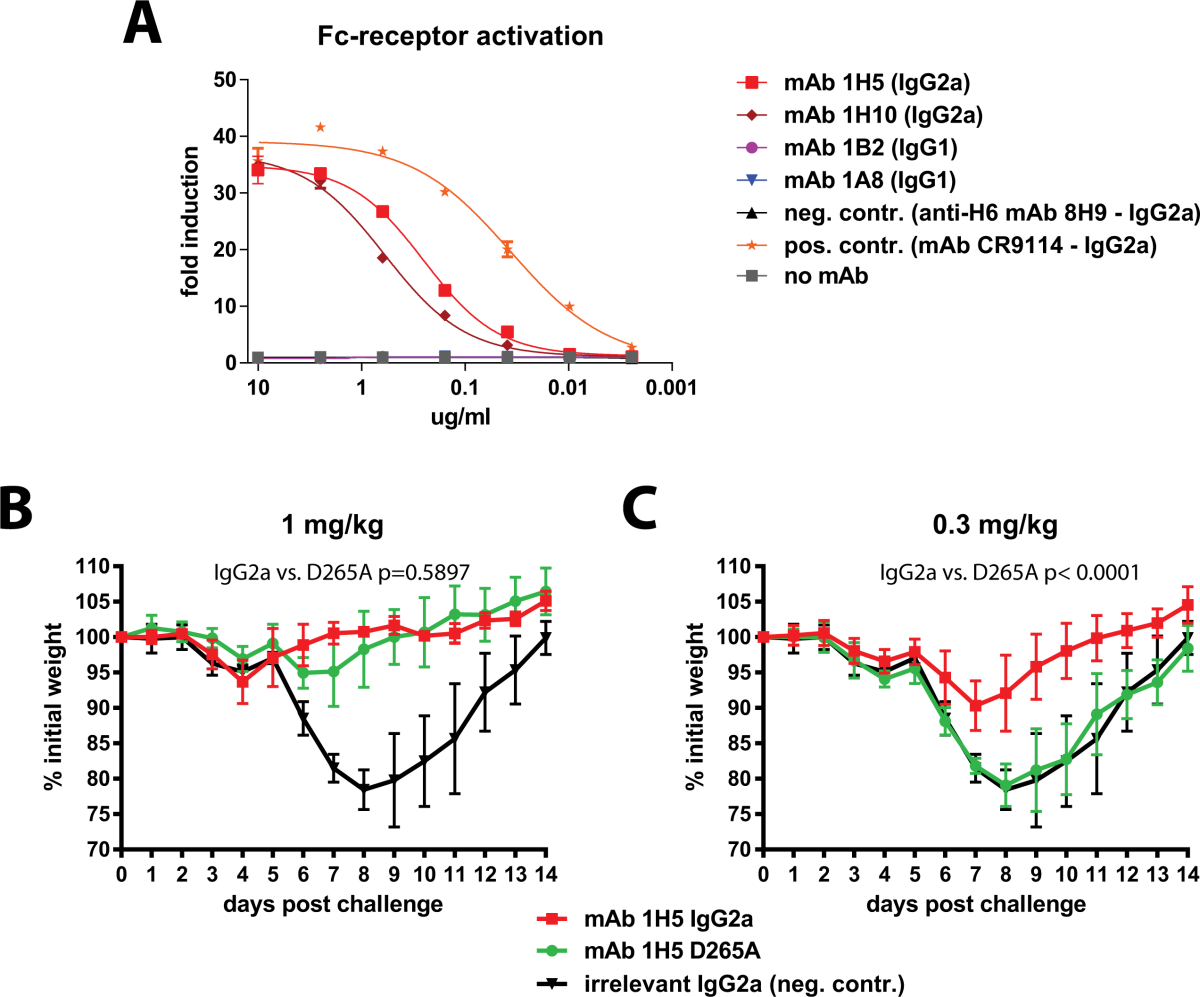
The protective effect of mAb 1H5 is partially dependent on its Fc-fragment. **A** shows activity of mAbs 1A8, 1B2, 1H5 and 1H10 in an Fc-receptor activation reporter assay. Only non-neutralizing antibodies 1H5 and 1H10 (both IgG2a) show activity in this assay. **A** recombinant mutant of mAb 1H5 that lacks interaction of its Fc-fragment (D265A, IgG2a) with Fc-receptors and complement was then compared to the recombinant IgG2a version of the mAb. **B** shows weight loss after challenge when mice were pre-treated with 1 mg/kg of mAb. **C** shows weight loss after pre-treatment with 0.3 mg/kg of mAb. Statistical analysis for comparison of wild type and D265A groups in **B** and **C** was performed using a 2-way ANOVA in Graphpad Prism. 5 mice per group were used for experiments shown in this figure.

MAbs 1H5 and 1H10 provide remarkable protection *in vivo* in the absence of appreciable *in vitro* neutralizing activity (Fig 3 and S2 Fig). Several mechanisms for antibody-mediated protection *in vivo* may contribute to this phenomenon: antibody-dependent cell mediated cytotoxicity (ADCC), antibody-dependent cellular phagocytosis (ADCP), antibody-dependent respiratory burst (ADRB) activity or complement-dependent cytotoxicity (CDC). These mechanisms rely on interactions of the antibody Fc-fragment with Fc-receptors or complement. First, we tested if mAbs 1A8, 1B2, 1H5 and 1H10 can activate Fc-receptor signaling. This was done using a commercial Fc-receptor activation reporter assay which measures activation of the murine FcyRIV. Both mAbs 1H5 and 1H10 showed activity in this assay while mAbs 1A8 and 1B2 were not active (most likely because the IgG1 isotype does not bind effectively to murine FcyRIV) (Fig 6A). We then assessed the role of Fc/Fc-receptor or Fc/complement interactions for protection *in vivo*. The 1H5 variable regions were cloned into expression vectors carrying murine IgG2a, IgG2a D265A and IgG1 frameworks. The D265A mutation disables interactions with Fc-receptors as well as complement [26]. The three variants of mAb 1H5 were then tested for antigen binding in an ELISA. While the IgG2a and IgG2a D265A variants showed very similar binding patterns the IgG1 variant lost binding at low antibody concentrations (S7A Fig). All three variants were tested in the Fc-receptor activation reporter assay and—as expected—only the wild type IgG2a version showed activity. Since the IgG1 version did not bind well as compared to the IgG2a and IgG2a D265A versions it was excluded from the main *in vivo* studies (but was added in a follow up experiment shown in S8 Fig and showed no protection at the dose tested). For the *in vivo* experiments mice received 1 or 0.3 mg/kg of 1H5 IgG2a, of 1H5 IgG2a D265A or of a control mAb (IgG2a) before challenge with Shanghai13 virus. No significant difference in weight loss was observed between wild type 1H5 and the D265A mutant at 1 mg/kg (Fig 6B). However, at 0.3 mg/kg the wild type 1H5 (IgG2a) was still protecting mice from weight loss while animals treated with 1H5 IgG2a D265A lost weight similar to the negative controls. In addition 1H5 had less protective effect in C3 KO mice than in wild type mice (S9 Fig). This data suggests that Fc/Fc-receptor or Fc/complement interactions may contribute to the protective effect of 1H5 at low concentrations.

## Discussion

The H7 subtype of influenza viruses has caused human infections in the past which has spurred the development of pre-pandemic influenza virus vaccines. The H7 vaccine trials demonstrated that live attenuated and inactivated H7 vaccines mostly failed to induce robust HI responses in humans [13, 16–18, 27] [11–13]. While the use of adjuvants increased HI titers significantly, the titers remained relatively low when compared to similar HI measurements taken after seasonal or even H5N1 vaccinations. A recent re-analysis of the immune response induced by an H7N1 vaccine showed that a strong immune response against the HA can be induced as measured by ELISA, even in the absence of a notable HI or MN responses [14]. Of note, the passive transfer of non-neutralizing human serum with strong reactivity to HA significantly reduced lung virus titers of H7N9 challenged mice [14]. This is reminiscent of the protective effect of non-neutralizing antibodies 1H5 and 1H10 that we have generated and characterized. MAb 1H5-mediated protection seems to be partially dependent on Fc/Fc-receptor or Fc/complement interactions in mice and non-neutralizing antibodies induced by H7 vaccination in humans could provide protection through the same mechanism. However, Fc/Fc receptor or Fc/complement interaction do not explain the protection seen at higher antibody concentrations with the D265A mutant antibodies. Alternative mechanisms, e.g mediated by interactions of the HA-bound mAb with natural defense proteins like surfactant protein D or mucins might play a role in the observed protection as well. This mechanism was recently described for antibodies that bind the human immunodeficiency virus (HIV) [28–30]. Notably, non-neutralizing but protective antibody responses would not be detected by standard influenza virus vaccine efficacy measurements such as traditional HI or *in vitro* neutralization assays. Importantly, the protective effect of highly neutralizing mAbs 1A8 and 1B2 in mice at the doses tested was comparable to the protective effect of non-neutralizing antibodies 1H5 and 1H10. This highlights the observation that *in vitro* neutralizing activity of antibodies may not always predict their prophylactic or therapeutic potency *in vivo*. This was also shown recently for stalk-reactive mAbs which gain much potency *in vivo* due to strong ADCC activity [31]. Similarly, H1N1 reactive but non-neutralizing antibodies were shown to be protective in a non-human primate model [32].

Two divergent H7 HA lineages, North American and Eurasian, primarily circulate in avian hosts. Both lineages have caused infections in humans [1, 33–35]. Furthermore, highly pathogenic avian H7 viruses emerged from both lineages and caused severe outbreaks in poultry in Europe and North America [33–36]. Our analysis of the epitope targets of mAbs 1A8 and 1B2 revealed that antigenic site A is highly conserved within and between the two lineages. All other putative antigenic sites do not show significant conservation between the Eurasian and North American lineages. This suggests the absence of immune pressure on site A in avian species or purifying selection. While it is unclear if site A would remain conserved during circulation in mammalian hosts it is important to note that site A escape mutants showed attenuation in the mouse model. The conservation of antigenic site A explains the cross-reactivity exhibited by our mAbs and the cross-reactivity found after H7 vaccination in animal models and humans [12, 14, 15, 19–21, 37–39]. The cross-reactivity exhibited by our mAbs is remarkable since the two lineages are genetically highly divergent and have not shared a common ancestor for many years. Moreover, their HAs share only 85% amino acid identity [20]. Although the HAs of seasonal H3N2 strains A/Perth/16/09 and A/Victoria/361/11 share 97.7% of their amino acids, the small 2.3% change resulted in an antigenic drift and made re-formulation of the vaccine necessary [20]. Given the conservation of the putative antigenic site A of H7 HAs it is likely that H7N9 vaccines that are currently developed could be protective in the case of future epidemic/pandemic outbreaks of H7 viruses.

## Materials and Methods

### Cells, viruses and recombinant proteins

Madin Darby canine kidney (MDCK, ATCC# CCL-34) cells were propagated in Dulbecco's Modified Eagles Medium (DMEM, Gibco) supplemented with 10% fetal bovine serum (FBS, HyClone) and antibiotics (penicillin-streptomycin mix, 100 units/ml of penicillin and 100 μg/ml of streptomycin, Gibco). The following viruses (described in [20]) were grown in 8–10 day old embryonated chicken eggs (Charles River Laboratories): A/Shanghai/1/13 (Shanghai13, H7N9, 6:2 re-assortant with internal genes from A/Puerto Rico/8/34 (PR8) virus), A/Shanghai/2/13 (H7N9, Shanghai2, 6:2 re-assortant with PR8), A/Anhui/1/13 (Anhui13, H7N9, 6:2 re-assortant with PR8), A/mallard/Netherlands/12/00 (mallNL00, originally H7N3, 7:1 re-assortant with PR8), A/rhea/North Carolina/39482/93 (rheaNC93, H7N1) and A/chicken/Jalisco/12283/12 (chickJal12, originally H7N3, 7:1 PR8 re-assortant, multi-basic cleavage site removed). Inoculated eggs were incubated at 37°C for 48 hours, chilled down to 4°C for 12 hours and allantoic fluids were harvested and clarified by low speed centrifugation. Viruses were titered by plaque assay on MDCK cells in the presence of tosyl phenylalanyl chloromethyl ketone (TPCK)-treated trypsin. To generate a substrate for enzyme-linked immunosorbent assays (ELISA), clarified allantoic fluid containing Shanghai13 virus was spun through a 30% sucrose cushion (in phosphate buffered saline (PBS), pH 7.4) in a Beckman ultracentrifuge (SW28 rotor, 25,000rpm, 2 hours at 4°C).

Sf9 insect cells (ATCC# CRL-1711) were propagated in TNM-FH medium (Gemini Bio-Products) supplemented with 10% FBS and antibiotics. *BTI-*TN5B1-4 cells (Vienna Institute of BioTechnology subclone [40]) were grown in serum free SFX medium (HyClone) supplemented with antibiotics. Recombinant baculoviruses expressing soluble versions of the H7 HAs of Shanghai13, Anhui13, mallNL00, BC04, chickJal12, chickIT99 viruses as well as of the PR8 HA, H15 HA (from A/shearwater/West Australia/2576/79, H15N9), chimeric H4/7 HA (cH4/7, H4 head domain from A/duck/Czech/56 on top of the stalk domain of Shanghai13 HA), the 1B2 and 1A8 escape mutant HAs and the trimeric globular head of Shanghai13 HA (all constructs are described in [14, 19]) were propagated in Sf9 cells. Recombinant baculoviruses were used to infect *BTI-*TN5B1-4 cells to express soluble recombinant proteins that were then purified from culture supernatants as described in a detailed protocol in [41, 42].

### Monoclonal antibody generation and purification

To generate H7 specific mAbs 6–8 week old female BALB/c mice were anesthetized (0.15 mg/kg ketamine and 0.03 mg/kg xylazine) and electroporated with 40 ug of pCAGGS plasmid expressing the Shanghai13 HA in the left calf muscle using a TriGrid electroporation device (Ichor Medical Systems). The procedure was performed twice in three week intervals. One mouse was then boosted with 100 ug (total protein) of Shanghai13 virus-like particles (produced as described in [14, 43]) adjuvanted with 10 ug polyI:C (Invivogen) via the intraperitoneal route (i.p.). Three days post boost the spleen was harvested and fused with SP2.0 cells as described in detail before [23]. Hybridoma colonies were grown in Medium D (Stemcell) semisolid media, single colonies were picked and cultivated in Medium E (Stemcell) media in 96-well plates. Culture supernatants were then screened for reactivity to recombinant Shanghai13 HA using a standard ELISA procedure as described below. Positive clones were isotyped and adapted and grown in Hybridoma-SFM (HyClone) serum free media. Antibody was then purified from 300–500 ml cultures via sepharose G columns using a standard protocol as described in detail before [23].

### Recombinant antibody generation and expression

To generate recombinant and mutant antibody constructs, RNA was extracted from a hybridoma cells using Trizol reagent (Invitrogen), according to manufacturer’s instructions. Variable heavy and kappa regions were amplified with gene-specific primers using SuperScript III One-Step RT-PCR with Platinum Taq (Invitrogen). Variable heavy and kappa regions were cloned into pFUSEss-CHIg-mG2a, pFUSEss-CHIg-mG1, or pFUSEss-CHIg-mG2a D265A and pFUSE2ss-CLIg-mK vectors, respectively (primer sequences available upon request). Human Embryo Kidney 293T cells were maintained in Dulbecco’s minimal essential medium (DMEM) supplemented with 10% fetal calf serum at 37˚C. Cells were transfected with corresponding heavy and light chains with PEI-max HCl (Polysciences, Inc.). Twentyfour hours post-transfection, media was replenished and supernatant was harvested 7 days post-transfection. Antibody supernatants were purified as described above.

### Western blots and ELISA

To assess if the four mAbs would react to denatured HA in Western blots, recombinant HA (rHA) proteins, Shanghai13 (H7) and PR8 (H1), were run on a 4–20% precast polyacrylamide gel (BioRad). Gels were washed in dH_2_O and transferred to a PVDF membrane via an Owl HEP Series Semidry Electroblotting system (Thermo Scientific), according to the manufacturer's instructions. Membranes were washed in PBS 1X and blocked with 10% non-fat milk-PBS for 2 hours at room temperature. Membranes were then washed three times with PBS 1X containing 0.1% Tween 20 (PBST). Antibodies of interest or a mouse anti-polyhistidine antibody (Sigma-Aldrich) (10 ug/ml) were incubated with the membrane for 1 hour at room temperature (RT) and washed three times in PBST. An anti-mouse IgG-horseradish peroxidase (HRP) conjugate was used for detection via a Western Lightinin Plus-ECL kit (Perkin Elmer) according to the manufacturer's instructions.

To assess binding of the four mAbs to divergent recombinant HA proteins and purified virus quantitative ELISA assays were performed. Immulon 4HBX Flat Bottom 96-well microtiter plates (Thermo Fisher Scientific) were coated overnight with recombinant HAs (2 ug/ml) or purified virus (5 ug/ml) in carbonate-bicarbonate coating buffer (0.1 M Na_2_CO_3_/NaHCO_3_, pH 9.4, 50 μL/well) at 4°C. Plates were then blocked using a 3% non-fat dry milk powder solution in PBS containing 0.1% Tween 20 (PBST) for 1 hour at room temperature (RT). After the blocking step, plates were incubated with 1:3 serial dilutions of the respective mAb in 1% milk PBST and incubated for 1 hour at RT (100 ul/well). Plates were washed three times with PBST (3x100 ul/well) and were then incubated for another hour with secondary anti-mouse HRP antibody (Rockland) at a dilution of 1:3000 in 1% milk PBST (50 ul/well). Plates were then again washed three times with PBST and developed using *o*-phenylenediamine dihydrochloride (SigmaFast OPD, Sigma) as substrate. After 10 minutes plates were stopped with 3M HCl, read at 490 nm on a Synergy 4 plate reader (BioTek) and the minimal binding concentration was calculated with a cut-off of blank reads plus 3x the standard deviation of the blank reads.

To measure the effect of the pH induced conformational change of the HA on antibody binding we performed ELISA assays with virus substrate that was pretreated with buffers of different pH. The procedure was similar to the ELISAs described above except that plates were washed twice with PBS after blocking and then incubated with the appropriate pH-buffered solutions (300 mM sodium citrate; pH 7.0, 5.8, 5.4, 5.0, 4.4, and 4.4 plus 0.1 M dithiothreitol (DTT)) for 30 minutes at RT before the antibody binding step.

### Competition ELISA

Nunc Maxisorp ELISA plates were coated with 50 μL of purified baculovirus-expressed H7 HA (2.5 μg/mL) and incubated overnight at 4°C. Plates were blocked with 5% non-fat milk/1X PBS for 30 minutes at RT and then incubated with 100 μL of mouse monoclonal antibodies (20 μg/mL) for 1 hour at RT. After incubation, plates were washed with 0.1% Tween 20/1X PBS (TPBS) three times. Four de-identified post-vaccination sera from a recent clinical trial with H7 vaccines were kindly provided by John Treanor, University of Rochester (ClinicalTrials.gov registration number NCT01534468) [20, 38, 44]. Human sera were initially diluted at 1:100 and serially diluted 2-fold across the plate in duplicates. After a one-hour incubation at RT, plates were washed three times again with TPBS and incubated with a secondary anti-human IgG-specific antibody conjugated to horseradish peroxidase (Millipore, Inc.). Plates were subsequently incubated at 37°C for 1 hour, washed three times with TPBS and developed with Sigmafast OPD peroxidase substrate (Sigma-Aldrich). The reaction was stopped with 50 μL of 3M HCl and absorbance was read at 492 nm. A non-linear regression curve was initially generated, an area under the curve was subsequently calculated using GraphPad Prism 6 and area reduction as compared to a control (no murine competing mAb) was calculated [45].

### Determination of binding kinetics

In order to determine K_D_ values, biolayer interferometry with the use of an Octet Red96 System (ForteBio) was performed, as described below. rHAs were biotinylated at a 1:1 molar ratio with the use of a “No Weigh” NHS-PEG4-Biotin (Thermo Scientific), according to the manufacturer's instructions. Biotinylated rHAs were loaded onto Dip and Read Streptavidin (SA) Biosensors (ForteBio) at a concentration of 20 ug/ml in 1X Kinetics Buffer (PBS 1X [pH 7.4], 0.002% Tween 20, 0.01% BSA) for 300 s. Following loading, a second baseline was monitored before association for 180 s. For measuring association (K_on_), the antibodies of interest were measured for 300 s by exposing sensors to eight concentrations of each antibody in 1X Kinetics Buffer. For measuring dissociation (K_off_), sensors were exposed to 1X Kinetics Buffer for 900 s. All of these steps were performed at 30°C. All eight sensor's readings were aligned to the dissociation and globally fit for determining the K_D_ values, with acceptable values having an R^2^ close to 1 and a *X* ^2^ under 3. If one or two sensors failed to have an R^2^ above 0.85, they were removed from calculation the global fit.

### Hemagglutination inhibition (HI) and microneutralization (MN) assay

The ability of mAbs to interfere with receptor binding was measured using an HI assay as described in [19]. Briefly, antibodies were serially diluted in PBS in 96-well V-bottom plates (Thermo Fisher Scientific) and then incubated at RT with 4 hemagglutination units of the respective virus (25 ul of diluted mAb plus 25 ul of virus). After 30 min of incubation 50 ul of a 0.5% turkey red blood cell solution (Lampire Biological Products) was added to the virus/mAb mixture and the assay was incubated for another 45 min at 4°C. MAbs were assessed in duplicate measurements. The highest concentration of mAb that was able to inhibit hemagglutination was reported as HI endpoint concentration.

To assess the *in vitro* neutralizing activity of the mAbs we performed a MN assay. Three-fold serially diluted antibody (starting at 300 ug/ml) in serum-free minimum essential medium (MEM) with TPCK-treated trypsin (Sigma-Aldrich) was mixed with an equal volume of virus (~1,000 TCID_50_) and incubated for 1 hour at 37°C. Monolayers of MDCK cells were washed with PBS and the virus/antibody mixture was added to the cells and incubated for 1 hour at 37°C. After the 1-hour infection procedure, the virus/antibody mixture was removed, and cells were cultured for 22 hours at 37°C with serum-free MEM containing TPCK-treated trypsin and the antibody at the appropriate concentration (to catch anti-viral effects downstream of entry). Cells were then washed with PBS 1X, fixed with 80% acetone at -20°C for 1 hour, washed three times with PBS 1X, blocked for 30 minutes with 5% non-fat milk- PBS, and then treated for 30 minutes with 3% H_2_O_2_. A biotinylated anti-nucleoprotein antibody (Millipore) diluted in 3% BSA (bovine serum albumin)-PBS was incubated for 1 hour at RT. A streptavidin-HRP conjugate was then added (100 ul/well). Plates were washed thrice and dried on paper towels. The plates were developed using SigmaFast OPD (100 ul/well) (Sigma-Aldrich), and the absorbance was measured at 490 nm on a Synergy 4 plate reader (BioTek).

### Passive transfer experiments in mice

To test the prophylactic *in vivo* protective effect of the mAbs, passive transfer experiments were performed. Six- to eight week old female BALB/c mice (n = 5 per group) were injected intraperitoneally with 1 or 5 mg/kg of mAbs 1A8, 1B2, 1H5 or 1H10. Control mice received an irrelevant mAb (mAb 8H9—specific for H6 HA) at 15 mg/kg. Naive mice were used as a second control group. Two hours post transfer mice were anesthetized (0.15 mg/kg ketamine and 0.03 mg/kg xylazine) and intranasally challenged with 10 murine lethal doses (mLD_50_) of Shanghai13 (10^4.24^ plaque forming units in 50 ul of PBS). Weight was monitored for 14 days and mice that lost 25% or more of their initial body weight were scored dead and euthanized according to institutional guidelines. MAbs 1B2 and 1H5 were also tested for their therapeutic potential. In this case mice were infected with 10 mLD_50_ first and were then treated with 10 mg/kg of mAbs (or control mAb 8H9) 48 or 72 hours post infection. To assess the reduction of lung virus titers animals received mAbs as described above and were then challenged with Shanghai13, mallNL00 or rheaNC93 viruses. Lungs were harvested on day 3 (3 mice per group) and on day 6 (3 mice per group) post infection, homogenized using a BeadBlaster24 (Benchmark) homogenizer and lung virus titers were assessed by plaquing lung homogenates on MDCK cells. In order to investigate the mechanism of protection of mAb 1H5, we also performed passive transfer challenge experiments (prophylactic, as described above) with recombinant mAbs 1H5 IgG2a (control) and 1H5 IgG2a D265A (Fc-null mutant) at 1 and 0.3 mg/kg. All antibodies for this experiment were recombinantly expressed in 293T cells.

To assess the impact of escape on viral fitness, the pathogenicity of the escape mutant viruses was evaluated in mice. Mice (n = 3–5 per group) were challenged with doses, ranging from 100 to 100,000 PFU, of the Shanghai13, Shanghai2, Shanghai2 1A8 escape, Shanghai2 1B2 escape or and Shanghai13 R65K escape mutant virus. Mice were monitored daily for clinical signs of morbidity and mortality up to 14 days post infection. Murine lethal dose 50 (LD_50_) concentrations were calculated using the Reed and Muench method.

In addition to the passive transfer experiments described above in BALB/c mice we also tested the protective effect of 1H5 (non-neutralizing) and 1B2 (neutralizing) in mice deficient for complement component C3 (B6.129S4-*C3* ^*tm1Crr*^/J, C3 KO, Jackson Laboratories) (n = 5–7 per group). Complement component C3 (in its activated form C3b) is an important part of the C5-convertase complex. C5-convertase activity is crucial for activating C5 which then initiates the formation of the membrane attack complex which mediates cell lysis. Importantly, only an intermediate phenotype is expected with C3 KO mice since thrombin is able to partially substitute the C5-convertase function in C3 KO mice [46]. Regular C57BL/6 mice were used as background controls and experiments were performed with 3 mg/kg of mAbs 1H5, 1B2 or 8H9 (control). For these experiments with C57BL/6 and C3 KO mice male and female animals were used with balanced proportions in all groups. LD_50_ values were determined for both C57BL/6 and B6.129S4-*C3* ^*tm1Crr*^/J but turned out to be highly similar (10^2.75^ and 10^3^ PFU respectively, S9 Fig).

All mouse experiments were performed in accordance with the guidelines of the Icahn School of Medicine at Mount Sinai Institutional Animal Care and Use Committee.

### Binding analysis by flow cytometry

293T cells were transfected pCAGGS plasmids coding for the Shanghai13 HA or Shanghai13 R65K (1H5/1H10 escape mutant) HA with the use of Lipofectamine 2000 (Life Technologies), according to manufacturer’s instructions. Forty-eight hours later, 293T cells were re-suspended and fixed with 4% para-formaldehyde for 30 minutes at RT. After fixing, cells were blocked with 5% non-fat milk/1X PBS for 1 hour at RT. Cells were washed with 1X PBS three times. MAbs of interested or H7-reactive polyclonal mouse serum were incubated with cells for 1 hour at RT at 5 ug/ml or 1:50 dilution, respectively. After incubation, cells were washed with 1X PBS three times. Primary antibodies were detected with a goat anti-mouse IgG Alexa Fluor 488-conjugated antibody (Life Technologies Inc.) at a 1:1000 dilution. Cells were washed with 1x PBS three times. Flow cytometry analysis was performed on a BD LSRII flow cytometer (BD Biosciences) and data was analyzed with FCSExpress (De Novo Software).

### Evolutionary analysis of antigenic sites

We used a phylogenetic framework to assess the diversity and distribution of epitopes across a globally sampled population of H7 subtype influenza viruses, including those that caused human infection. We downloaded all available H7-HA gene sequences from GenBank, excluding those less than 900 base pairs in length. We then created a multiple sequence alignment using the default settings in MUSCLE [47]. The sequence alignment was manually optimized. The 3’ and 5’ non-coding ends were trimmed and sequences with point insertions or deletions resulting in frame-shifts or premature stop codons were removed. The final matrix was comprised of 1468 gene sequences and 1710 base pairs.

The multiple sequence alignment was then translated allowing for pairwise comparisons of putative antigenic sites A-E (7 coding regions) [37, 48, 49]. Each coding region was considered a discrete unit. We therefore identified and assigned a discrete category for each antigenic site variant/sequence alignment. This allowed us to rapidly identify the sequence variant at each antigenic site and assess the lineage specific frequency of each antigenic site variant in the phylogeny.

The purpose of this tree reconstruction is to determine the frequency of antigenic site variants in the major lineages. We therefore, used a neighbor joining, distance based method to reconstruct the phylogenetic history. We optimized the distance estimates by applying a Hasegawa Kishino Yano (HKY) nucleotide substitution model. This method allows for the rapid and statistically consistent identification of major lineages. The Eurasian Avian and the North American Avian lineages could be robustly recovered in this analysis. Based on this delineation we could enumerate the lineage specific proportion of antigenic site variants.

### Epitope mapping

To generate *in vitro* escape mutants for neutralizing mAbs 1A8 and 1B2 10^6^ PFU of Shanghai2 or rheaNC93 viruses were incubated at RT for one hour with a neutralizing concentration (100 ug/ml) of mAb in a volume of 100 ul. The mixture was then injected into 10 day old embryonated chicken eggs and virus was harvested as described above. Virus was then plaqued on MDCK cells in the presence of neutralizing concentrations of the respective mAb, plaques were picked and grown up in eggs. RNA was extracted from the allantoic fluid using Trizol reagent (Invitrogen) according to the manufacturer's instructions. The RNA was then reverse transcribed with HA specific primers (sequence available upon request) and the Superscript III kit (Invitrogen) according to the manufacturer's instructions. cDNA was then amplified via polymerase chain reaction and sequenced by standard Sanger sequencing. Since mAbs 1H5 and 1H10 have no neutralizing activity the generation of escape mutants is not easily possible. We therefore screened a panel of H7 HA mutants that we previously generated (with 11 human H7-reactive monoclonal antibodies) for reduced binding of 1H5 and 1H10 by immuno-fluorescence microscopy as described in [50].

### Fc-receptor activation reporter assay

To assess if antibody-dependent cell-mediated cytotoxicity (ADCC) activity can be induced for non-neutralizing antibodies, an Fc-receptor activation reporter bioassay with effector cells expressing murine FcyRIV (Promega) was used, according to manufacturer’s instructions. MDCK cells were infected at a multiplicity of infection of three with Shanghai13 for one hour. Cells were washed, replenished with 1X MEM in the absence of trypsin, and incubated at 37˚C for 16 hours. Cells were then incubated with antibodies of interest for 30 minutes at 37˚C. Effector cells expressing murine FcyRIV were then added to the infected layer of MDCKs and incubated for six hours at 37˚C. Then the luminescence signal produced by the expression of firefly luciferase in the reporter cells was measured and the induction of signal above baseline was calculated.

### Ethics statement

All animal experiments were approved by the Icahn School of Medicine at Mount Sinai Institutional Animal Care and Use Committee (protocol number IACUC-2014-0255). Animals were housed and maintained in accordance with the US Animal Act PL99-158 and guidelines stated in the “Guide for the Care and Use of Laboratory Animals”. Embryonated eggs used in this study were inoculated between days 8 and 10 of embryonation, allantoic fluid was harvested between days 10 and 12 of embryonation.

## Supporting Information

S1 Fig **Binding kinetics of mAbs 1A8 (A), 1B2 (B), 1H5 (C), 1H10 (D) and negative control antibody 8H9 (E).** Binding was assessed on an Octet Red device. Binding was assessed at concentrations of 200, 66.6, 22.2, 7.41, 2.47. 0.823 and 0.274 nM.
(TIF)

S2 Fig *In vitro* neutralization activity of 1B2, 1A8, 1H5 and 1H10 against Shanghai13. (TIF)

S3 Fig H7-reactive mAb FAE1 mediates no protection against Shanghai13 challenge. MAb FAE1 (IgG2a, K_D_ 6.93x10^-9^) shows affinity in the range of neutralizing mAb 1B2 (IgG1, K_D_ 1.38x10^-10^) and 1H5 (IgG2a, K_D_ 4.75x10^-10^) but has no protective effect against H7N9 challenge in mice. Numbers in the figure legend indicate proportion of surviving mice (surviving/dead).
(TIF)

S4 Fig Binding of mAbs 1H5 and 1H10 to cells transfected with wild type H7 HA or the R65K mutant. Binding of polyclonal anti-H7 mouse serum (**A**), mAb 1H5 (**B**) or mAb 1H10 (**C**) or a negative control mAb (6F12 [23]) (**D**) to wild type H7 HA, R65K H7 HA or mock transfected cells.
(TIF)

S5 Fig Quantitative analysis of loss of binding of mAbs 1H5 and 1H10 to the R65K escape mutant. **A** and **B** show binding of polyclonal serum and 1H5 and 1H10 respectively to cells expressing the wild type Shanghai13 HA. **C** and **D** show binding of the same antibodies to cells transfected with the R65K escape H7 HA. A clear shift to the left for 1H5 and 1H10 binding to the R65K mutant is visible as compared to the wild type HA. This data corresponds well with the immunostaining phenotype shown in S4 Fig.
(TIF)

S6 Fig Non-neutralizing mAbs 1H5 and 1H10 compete with human post-H7 vaccination serum for binding to H7 HA. This indicates that H7 vaccination in humans induced antibodies that bind to epitopes that are similar or identical to the epitopes of 1H5 and 1H10. A murine control mAb of the same isotype did not show competition.
(TIF)

S7 Fig Characterization of 1H5 recombinants. **A** ELISA binding to H7 HA. The IgG1 version of mAb 1H5 shows lower binding as compared to the IgG2a and IgG2a D265A variants. **B** Activity in an Fc-receptor activation assay. Only the wild type IgG2a variant of mAb 1H5 shows activity.
(TIF)

S8 Fig The IgG1 version of mAb 1H5 loses its protective effect *in vivo*. Recombinantly expressed mAbs (1H5 as IgG1, IgG2a, IgG2a D265A and negative control antibody 8H6) were passively transferred into mice (n = 4–5 per group) via the i.p. route. Mice were then challenged with 10 LD_50_ of Shangai13 H7N9 virus. Numbers in the figure legend indicate proportion of surviving mice (surviving/dead).
(TIF)

S9 Fig Protective effect of non-neutralizing mAb 1H5 and neutralizing mAb 1B2 in C3 KO mice. **A** Comparative weight loss and survival of wild type C57BL mice and C3 KO mice after challenge with 100 plaque forming units of Shanghai13 (n = 3 per group). Wild type (C57B or B6) or C3 KO mice were administered a dose of 3 mg/kg of 1B2 or 1H5 via the i.p. route. Weight loss is shown in **B** (wild type) and **D** (C3 KO), survival is shown in **C** (wild type) and **E** (C3 KO). 5 to 7 mice per group (mixed sexes, sex balanced) were used for experiments shown in **B-E**. Weight loss of wild type (C57B or B6) **F** or C3 KO **G** mice administered a dose of 10 mg/kg of control mAb, 1B2 or 1H5 via the i.p. route. **H** Lung titers on day 3 post infection. 3–7 mice per group were used in panels **F-H**. Weight loss of 1H5 and 1B2 groups was compared using an unpaired t-test in Graphpad Prism.
(TIF)

S1 Table Antigenic site sequence distribution in Eurasian Lineage isolates. (DOCX)

S2 Table Antigenic site sequence distribution in North American Lineage isolates. (DOCX)

S3 Table Antigenic site sequence distribution in recent H7 isolates. (XLSX)

S4 Table LD_50_ values of wild type viruses and escape mutants in BALB/c mice. (DOCX)
