# Supplementary figures and images for: Broadly-Reactive Neutralizing and Non-neutralizing Antibodies Directed against the H7 Influenza Virus Hemagglutinin Reveal Divergent Mechanisms of Protection
Source: PLoS Pathog. 2016 Apr 15;12(4):e1005578. doi: 10.1371/journal.ppat.1005578 (PMC4833315; doi:10.1371/journal.ppat.1005578)

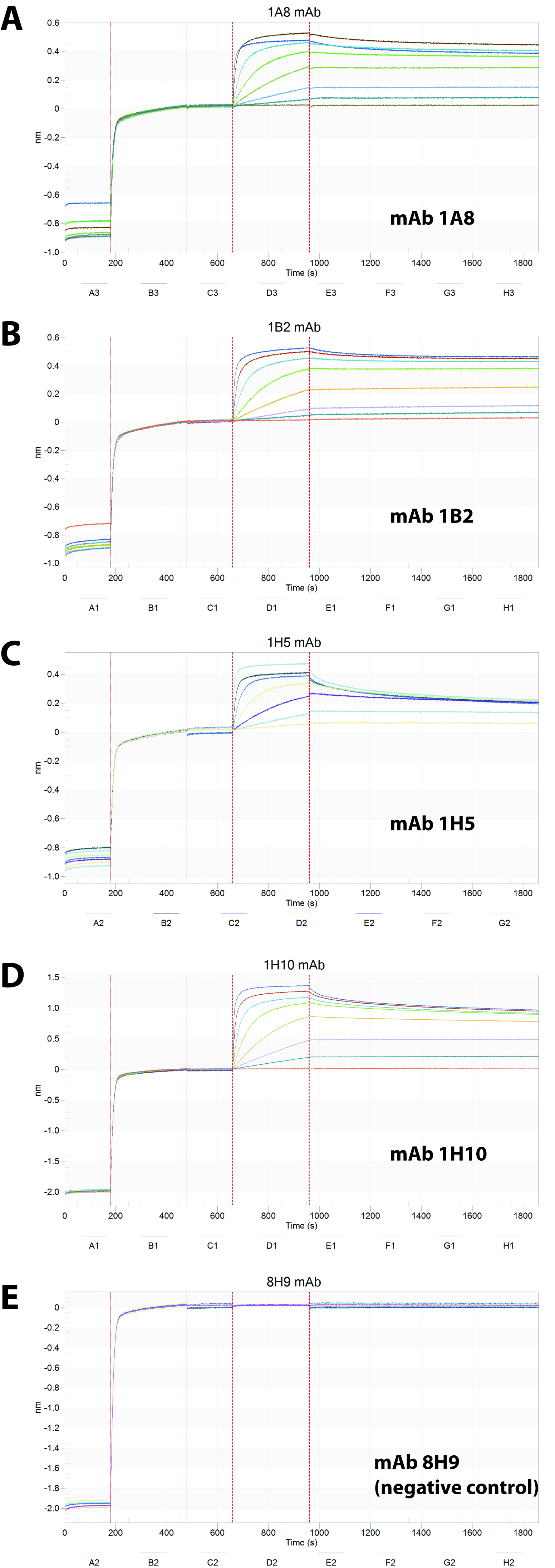

Supplement: S1 Fig — Binding kinetics of mAbs 1A8 (A), 1B2 (B), 1H5 (C), 1H10 (D) and negative control antibody 8H9 (E). Binding was assessed on an Octet Red device. Binding was assessed at concentrations of 200, 66.6, 22.2, 7.41, 2.47. 0.823 and 0.274 nM. (TIF) [file ppat.1005578.s001.tif]

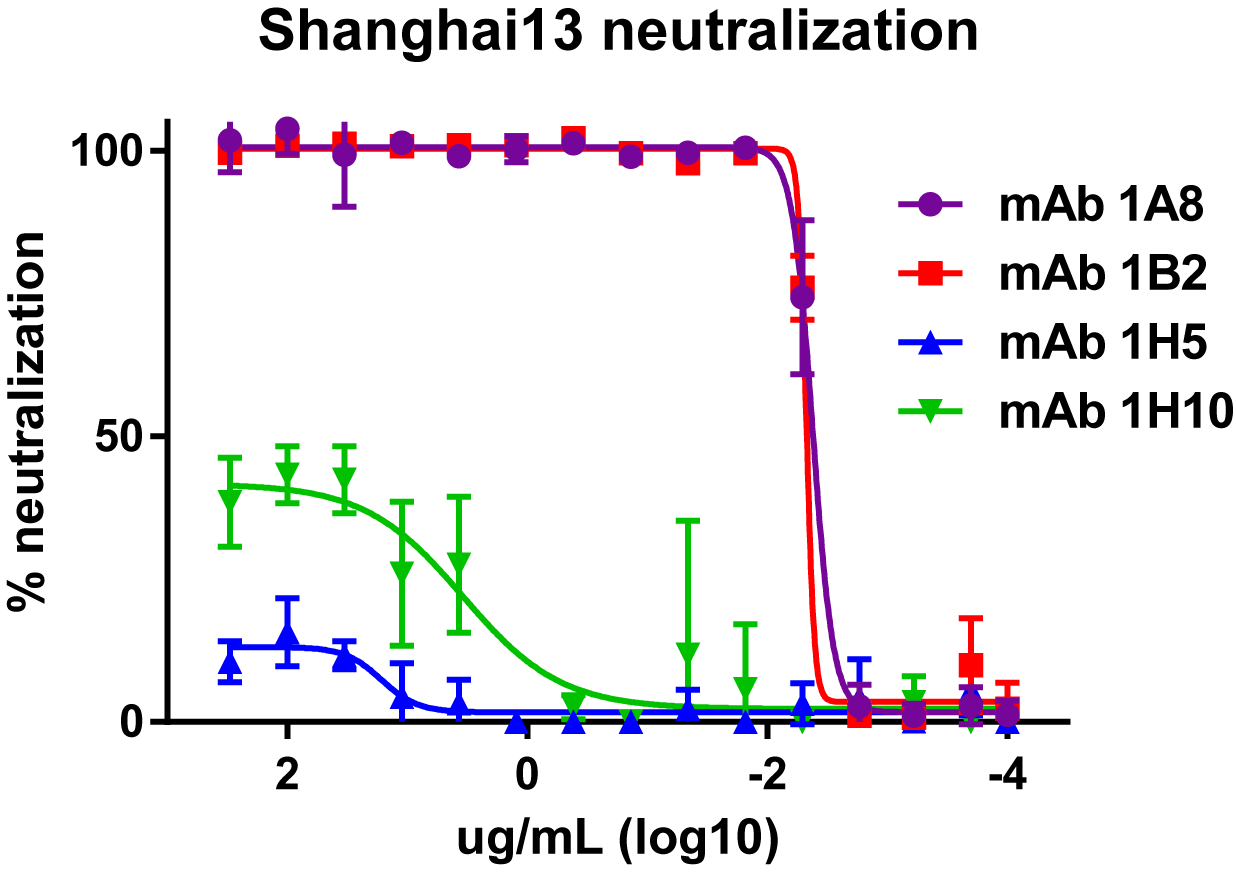

Supplement: S2 Fig — (TIF) [file ppat.1005578.s002.tif]

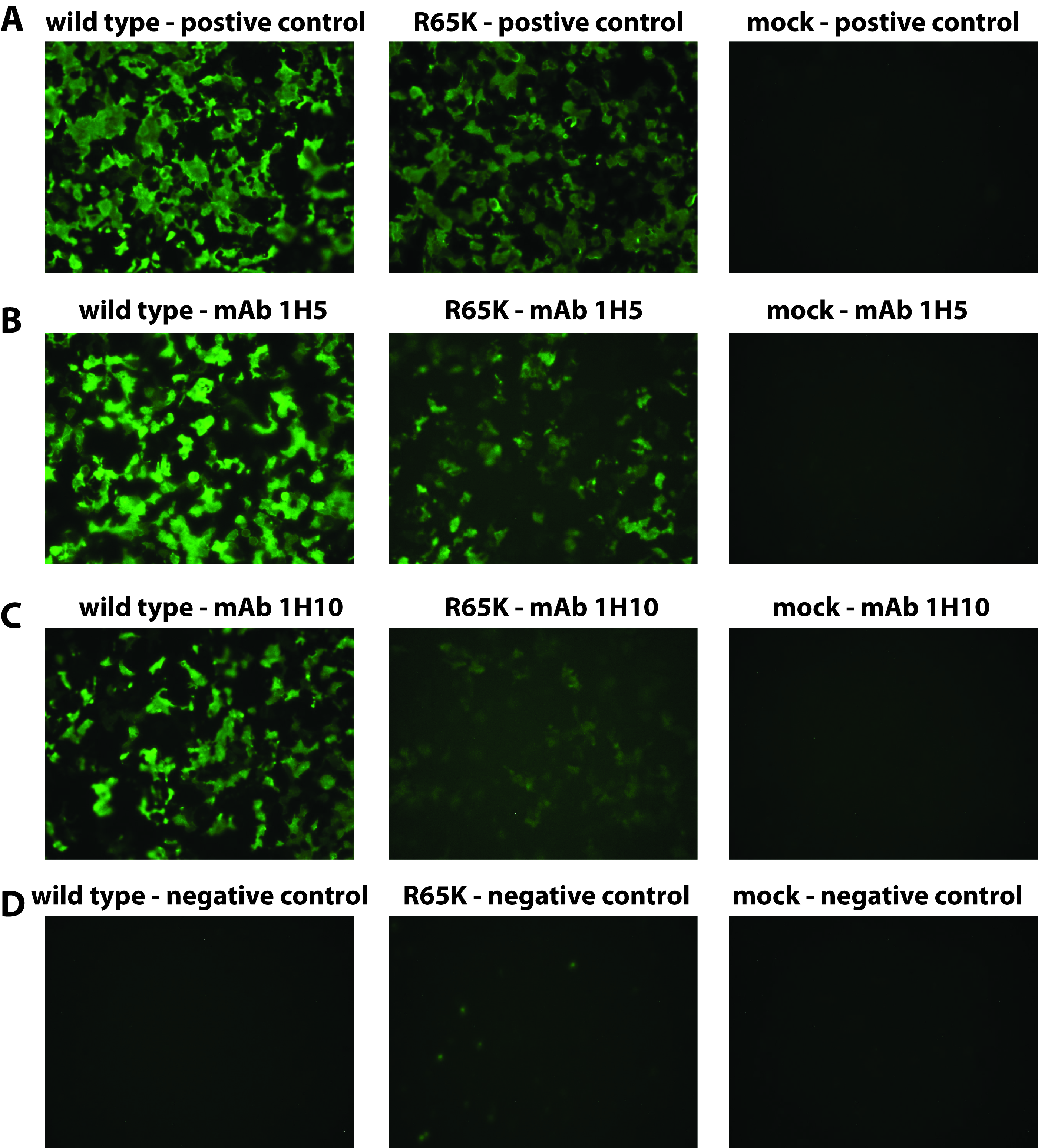

Supplement: S4 Fig — Binding of polyclonal anti-H7 mouse serum (A), mAb 1H5 (B) or mAb 1H10 (C) or a negative control mAb (6F12 [23]) (D) to wild type H7 HA, R65K H7 HA or mock transfected cells. (TIF) [file ppat.1005578.s004.tif]

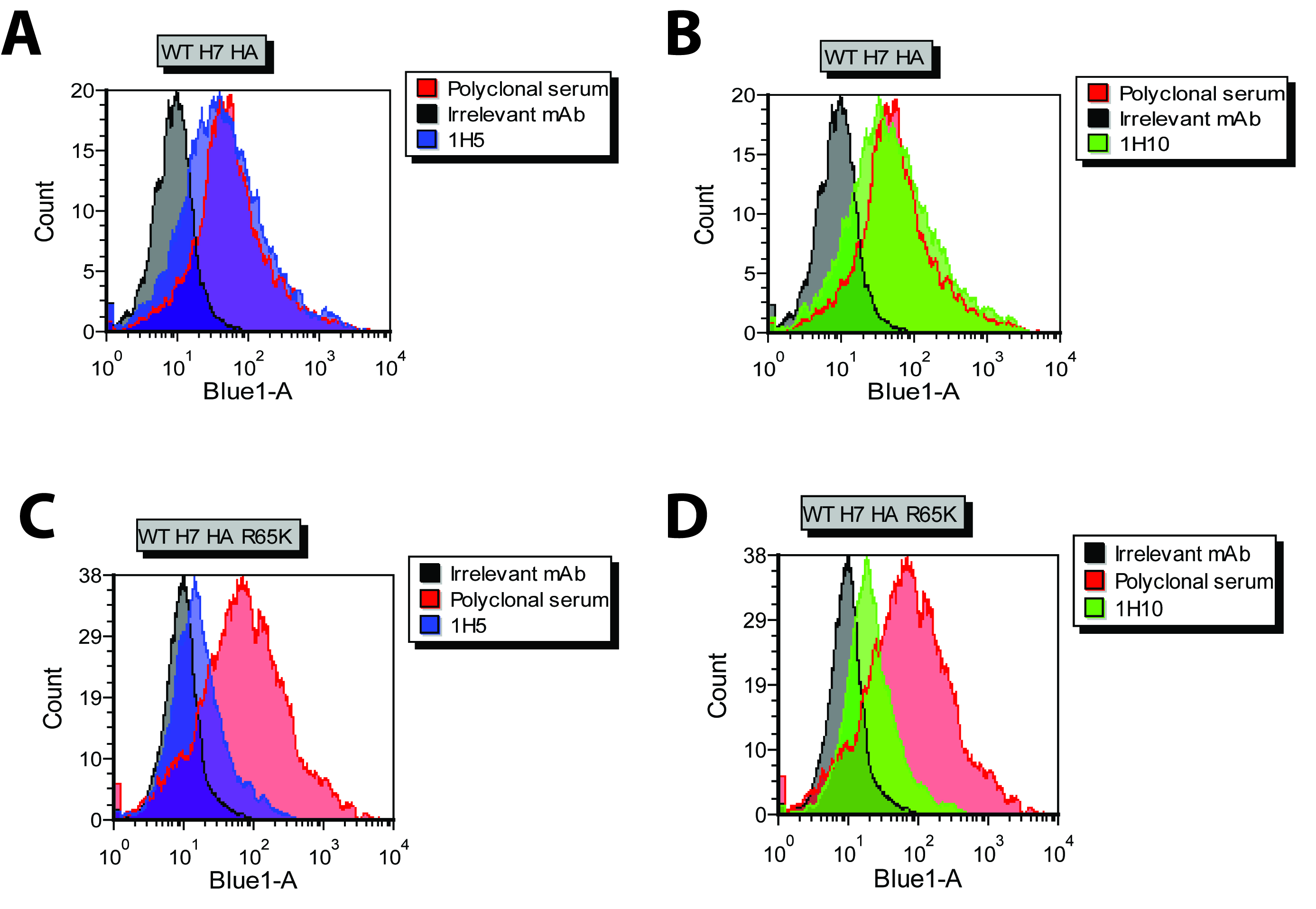

Supplement: S5 Fig — A and B show binding of polyclonal serum and 1H5 and 1H10 respectively to cells expressing the wild type Shanghai13 HA. C and D show binding of the same antibodies to cells transfected with the R65K escape H7 HA. A clear shift to the left for 1H5 and 1H10 binding to the R65K mutant is visible as compared to the wild type HA. This data corresponds well with the immunostaining phenotype shown in S4 Fig. (TIF) [file ppat.1005578.s005.tif]

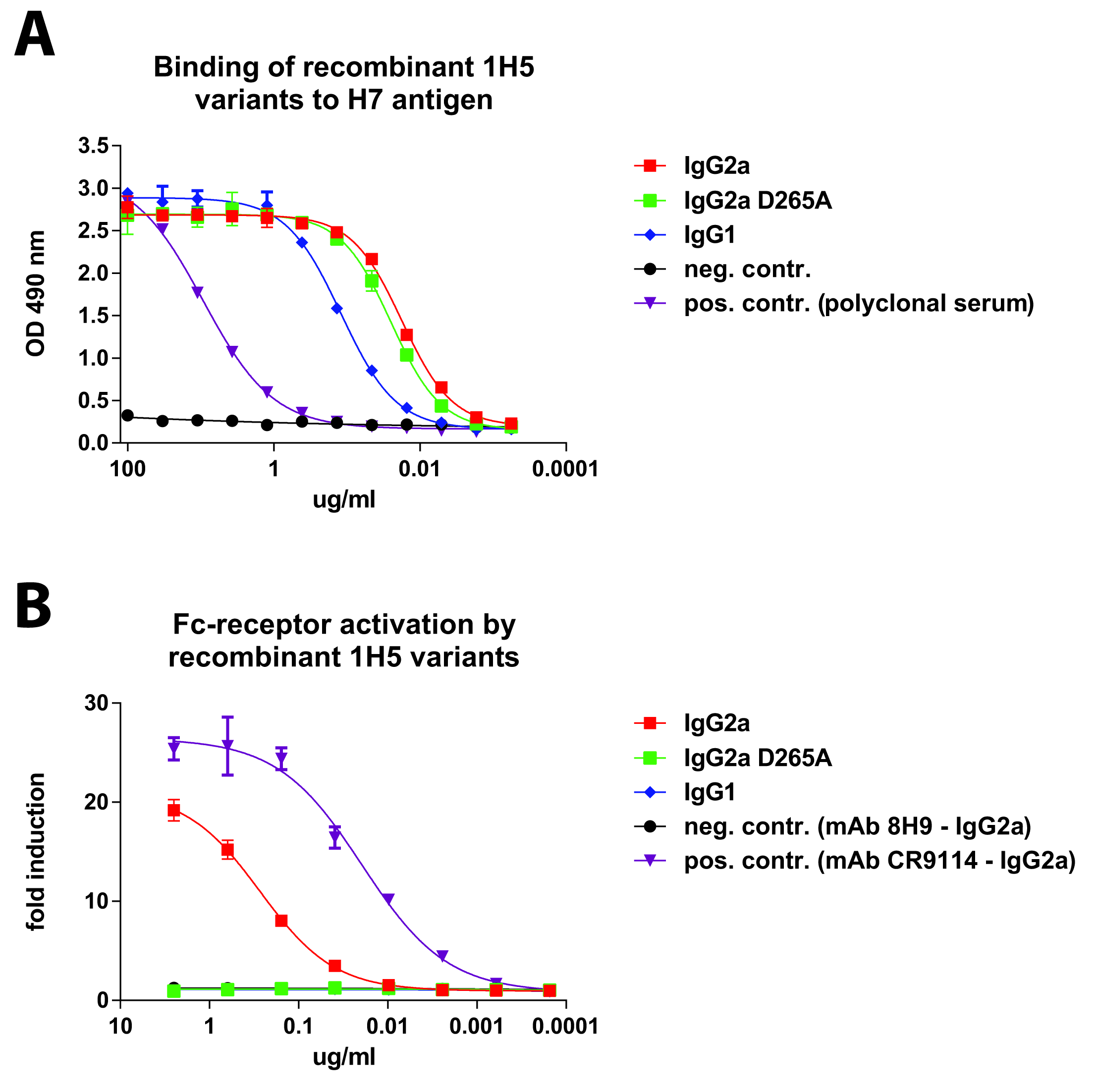

Supplement: S7 Fig — A ELISA binding to H7 HA. The IgG1 version of mAb 1H5 shows lower binding as compared to the IgG2a and IgG2a D265A variants. B Activity in an Fc-receptor activation assay. Only the wild type IgG2a variant of mAb 1H5 shows activity. (TIF) [file ppat.1005578.s007.tif]

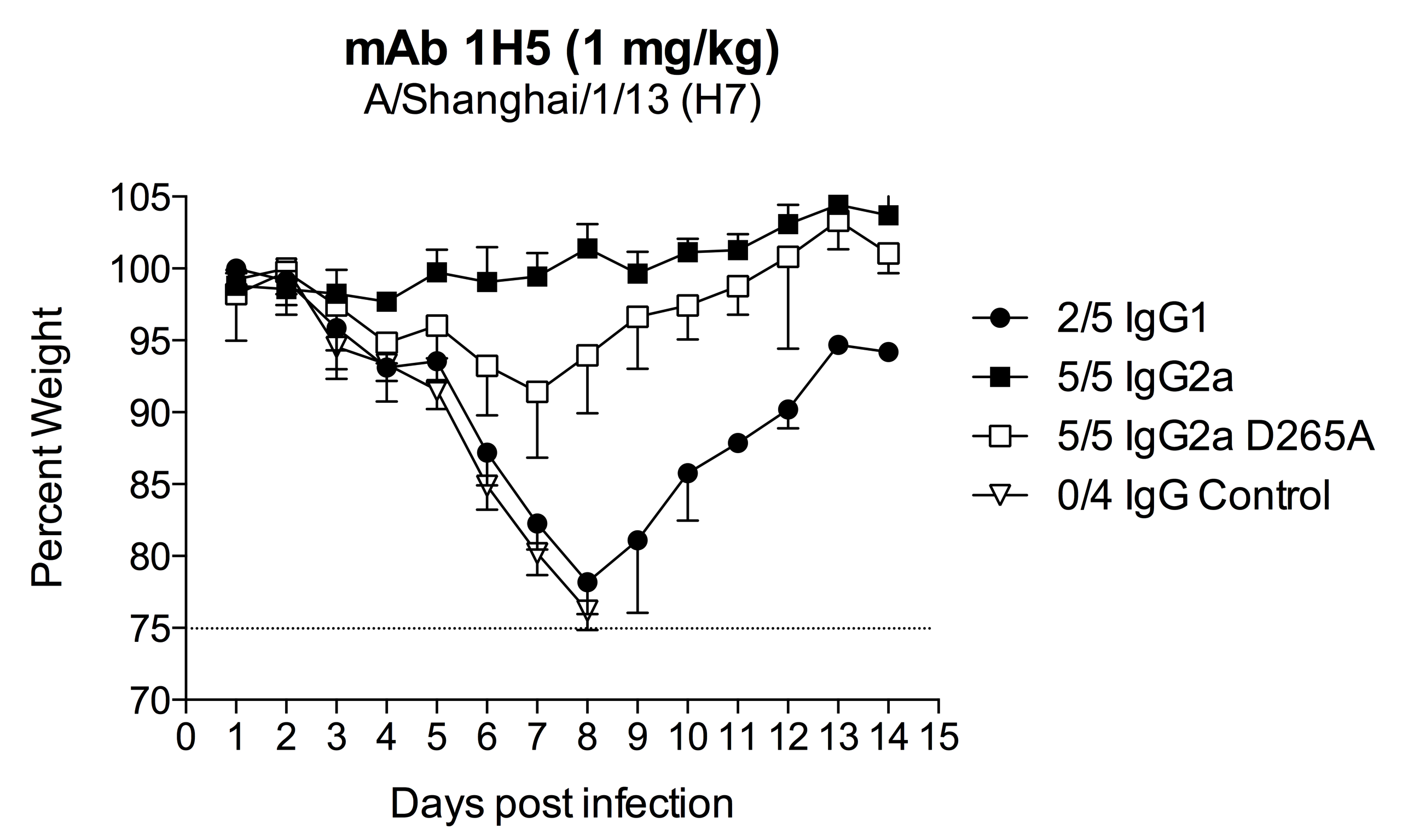

Supplement: S8 Fig — Recombinantly expressed mAbs (1H5 as IgG1, IgG2a, IgG2a D265A and negative control antibody 8H6) were passively transferred into mice (n = 4–5 per group) via the i.p. route. Mice were then challenged with 10 LD50 of Shangai13 H7N9 virus. Numbers in the figure legend indicate proportion of surviving mice (surviving/dead). (TIF) [file ppat.1005578.s008.tif]

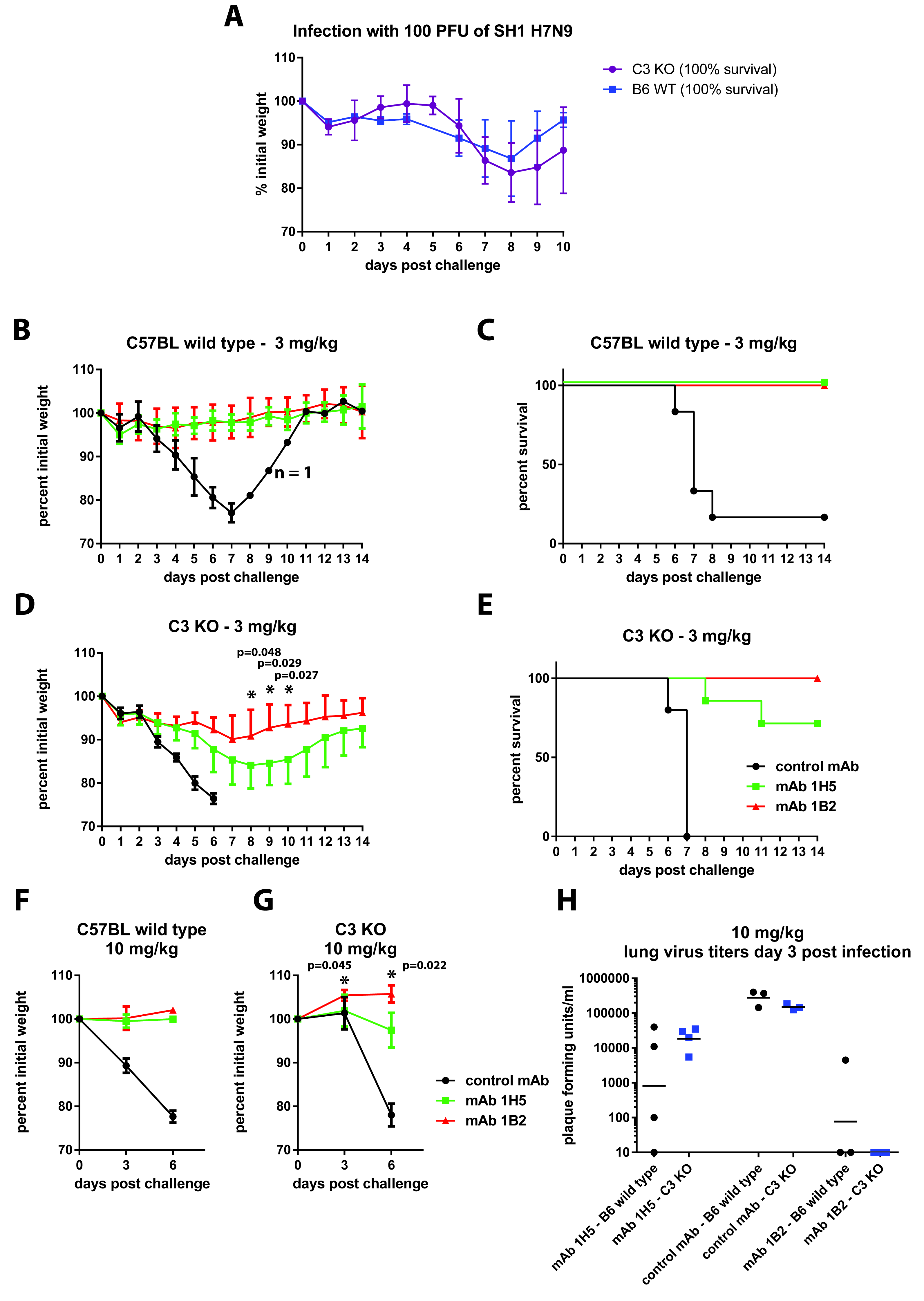

Supplement: S9 Fig — A Comparative weight loss and survival of wild type C57BL mice and C3 KO mice after challenge with 100 plaque forming units of Shanghai13 (n = 3 per group). Wild type (C57B or B6) or C3 KO mice were administered a dose of 3 mg/kg of 1B2 or 1H5 via the i.p. route. Weight loss is shown in B (wild type) and D (C3 KO), survival is shown in C (wild type) and E (C3 KO). 5 to 7 mice per group (mixed sexes, sex balanced) were used for experiments shown in B-E. Weight loss of wild type (C57B or B6) F or C3 KO G mice administered a dose of 10 mg/kg of control mAb, 1B2 or 1H5 via the i.p. route. H Lung titers on day 3 post infection. 3–7 mice per group were used in panels F-H. Weight loss of 1H5 and 1B2 groups was compared using an unpaired t-test in Graphpad Prism. (TIF) [file ppat.1005578.s009.tif]
