# Supplementary material for: Broadly-Reactive Neutralizing and Non-neutralizing Antibodies Directed against the H7 Influenza Virus Hemagglutinin Reveal Divergent Mechanisms of Protection
Source: PLoS Pathog. 2016 Apr 15;12(4):e1005578. doi: 10.1371/journal.ppat.1005578 (PMC4833315; doi:10.1371/journal.ppat.1005578)
Supplement: S1 Table — (DOCX) [file ppat.1005578.s010.docx]

| Site-A | Site-B1 | Site-B2 | Site-D | Site-E1 | Site-E2 | Site-C |
| --- | --- | --- | --- | --- | --- | --- |
| RRSGSS (83.3) | WLLSNTDNAA (78.8) | TTEQTKLYGS (61.4) | VGSSNYQQSFVPSPGAR (71.6) | NTRKD (64.3) | FLRGKS(95.0) | NCEGDC(77.5) |
| RRSRSS (4.9) | WLLSNTNNAA (7.4) | TAEQTKLYGS (23.7) | VGSSNYQQSFVPSPGER (10.5) | NTRKS (13.9) | FLKGKS(2.1) | SCEGDC(11.9) |
| ARSGSS (2.4) | WLLSNNDNAA (2.9) | TTEQTRLYGS (4.6) | VRSSNYQQSFVPSPGAG (3.6) | NTRRD (5.0) | FFKGKS(1.7) | NCEGEC(5.7) |
| KRSESS (2.3) | WLLSNTDNGV (1.6) | TTEQTKLYGG (2.7) | VESSNYQQSFVPSPGAR (3.3) | NTRKG (4.1) | FFKGES(0.5) | GCEGNC(1.9) |
| RRSGPS (2.1) | WLLSNNDNDA (1.5) | TAEQTRLYGS (1.5) | VGSSNYQQSFVPSLGAR (1.3) | NTRKA (3.6) | FLRGRS(0.4) | NCEGNC(1.1) |
| RRPGSS (1.9) | WLLSSTDNAA (1.3) | AAEQTKLYGS (1.2) | VGSSNYQQSFVPSPEAR (1.2) | NTRNE (2.3) | FLRGES(0.3) | DCEGDC(0.8) |
| WRSGSS (1.1) | WLLSNSDNAA (0.9) | TTEQAKLYGS (1.2) | VGSSNYHQSFVPSPGAR (1.1) | NTKRE (1.6) |  | SCKGDC(0.7) |
| RRSESS (0.6) | WLLSDTDNAA (0.8) | ATEQTKLYGS (0.9) | VWSSKYQQSFAPNPGPR (0.8) | NIRKD (1.3) |  | NCDGDC(0.3) |
| KRSGSS (0.5) | WLLSNKDNAA (0.7) | TTEQIKLYGS (0.7) | VGSSNYQQSFVPSPGSR (0.7) | NTRRE (0.9) |  | NCGGDC(0.3) |
| QRSGSS (0.3) | WLLSNADNAA (0.7) | VTEQIKLYGN (0.5) | VGSSNYQQSFIPSPGAR (0.7) | NTRND (0.8) |  |  |
| RRSSSS (0.3) | WLLSNNDNVA (0.5) | STEQTKLYGS (0.5) | VGSSKYHQSFVPSPGTR (0.7) | NSRKD (0.5) |  |  |
| SRSGSS (0.3) | WLLSNSDNVA (0.5) | NAEQTKLYGS (0.4) | VESSNYQQSFVPSPGVR (0.7) | NKRRE (0.4) |  |  |
|  | WLLSNADNAT (0.5) | TTEQTKLYGN (0.3) | IGSSKYQQSFTPSPGAR (0.5) | NTKKV (0.4) |  |  |
|  | WLLSNSDNAT (0.4) | ITEQTKLYGS (0.3) | VASSNYHQSFSPSPGAR (0.5) | NTRRG (0.3) |  |  |
|  | WLLSNSDNSA (0.4) | ATEQTKLYGN (0.1) | VGSSNYLQSFVPSPGAR (0.4) | NTKKD (0.3) |  |  |
|  | WLLSNTDNAT (0.4) |  | VGSSNYRQSFVPSPGAR (0.4) | NTRED (0.3) |  |  |
|  | WLLSNKDNDA (0.3) |  | VGSSKYQQSLVPSPGAR (0.4) |  |  |  |
|  | WLLSNKENAT (0.3) |  | VWSSKYQQSFVPNPGPR (0.4) |  |  |  |
|  | WLLSNSDNAV (0.1) |  | VWSSKYQRSFAPSPGPR (0.4) |  |  |  |
|  |  |  | VWSSKYQQSFAPSPGPR (0.4) |  |  |  |
|  |  |  | VGSSKYQQSFVPNPETR (0.3) |  |  |  |
|  |  |  | VGSSNYQQSFIPSPEAR (0.3) |  |  |  |

Eurasian Lineage Antigenic Site Variants. Variant (Percent prevalence)
