# Supplementary material for: Broadly-Reactive Neutralizing and Non-neutralizing Antibodies Directed against the H7 Influenza Virus Hemagglutinin Reveal Divergent Mechanisms of Protection
Source: PLoS Pathog. 2016 Apr 15;12(4):e1005578. doi: 10.1371/journal.ppat.1005578 (PMC4833315; doi:10.1371/journal.ppat.1005578)
Supplement: S2 Table — (DOCX) [file ppat.1005578.s011.docx]

| Site-A | | Site-B1 | | Site-B2 | | Site-D | Site-E1 | | Site-E2 | | Site-C | |
| --- | --- | --- | --- | --- | --- | --- | --- | --- | --- | --- | --- | --- |
| RRSGSS (49.4) | | WLLSNSDNAA (80.4) | | VSEQTKLYGS (45.0) | | VGSSKYQQSFTPSPGAR (51.8) | NPRNK (93.4) | | FFRGES (93.4) | | GCEGDC (31.1) | |
| TRSGSS (38.1) | | WLLSNSDNSA (11.8) | | ATEQTKLYGS (31.5) | | VRSSKYQQSFTPSPGAR (29.1) | NPRDK (2.5) | | FLRGES (2.0) | | SCGGDC (24.2) | |
| KRSGSS (8.0) | | WLLSSSDNAA (2.0) | | ADEQTKLYGS (6.6) | | VRSSKYQQSFTPNPGAR (7.4) | NPRSK (1.3) | | FFKGES (1.5) | | SCRGDC (23.1) | |
| RRLGSS (2.0) | | WLLSNSDNAT (1.8) | | ANEQTKLYGS (4.6) | | VISSKYRQSFTPSPGAR (2.1) | NPKSK (1.0) | | FLKGKS (1.0) | | SCGGNC (6.0) | |
| SRSGSS (0.6) | | WLLSNTDNAA (1.5) | | TTEQTKLYGS (4.5) | | VRSSKYQQSFTPSPGTR (1.8) | NPKDK (0.7) | | FFRGKS (0.8) | | GCEGNC (5.9) | |
| RRLGPS (0.3) | | WLLSNSNNAA (1.3) | | VSEQTKLYGN (2.2) | | VRSSKYQQSFTPSTGAR (1.5) | NSRNK (0.6) | | FFRGAS (0.6) | | NCGGDC (2.0) | |
| QRSGSS (0.3) | | WLLSNSDNAV (0.8) | | ASEQTKLYGS (1.7) | | VGSSKYQQSFTPSPGTR (1.3) | NPKNK (0.3) | | FFRGGS (0.4) | | GCGGDC (1.5) | |
| ARSGSS (0.3) | | WLLSNSDNVA (0.4) | | AAEQTKLYGS (1.0) | | VKSSKYQQSFTPSPGAR (1.0) | NPRKK (0.3) | | FLRGKS (0.3) | | SCEGDC (1.3) | |
| RRSDSS (0.3) | |  | | TAEQTKLYGS (0.7) | | VESSKYQQSFTPSPGAR (0.7) |  |  |  |  | SCGGGC (1.3) | |
| RRSSSS (0.3) | |  | | ISEQTKLYGS (0.6) | | VRSSKYQQSFAPSPGAR (0.6) |  |  |  |  | NCVGDC (1.0) | |
| RRPGSS (0.3) | |  | | ANEQTRLYGS (0.4) | | IGSSKYQQSFTPSPGAR (0.4) |  |  |  |  | NCRGDC (1.0) | |
| RRTGSS (0.1) | |  |  | TTEQTRLYGS (0.4) | | IRSSKYQQSFTPSPGAR (0.4) |  |  |  |  | NCGGNC (0.6) | |
| RRSGPS (0.1) | |  |  | VTEQTKLYGS (0.3) | | VGSSKYQQSFIPSPGAR (0.4) |  |  |  |  | GCEGGC (0.4) | |
|  |  |  |  | DTEQTKLYGS (0.3) | | VGSSKYQQSFTPSPEAR (0.3) |  |  |  |  | ACEGDC (0.3) | |
|  |  |  |  | TTEQTKLYGN (0.1) | | VGSSKYQQSFTPIPGAR (0.3) |  |  |  |  | SCGGEC (0.3) | |
|  |  |  |  | ATEQTKLYGN (0.1) | | VRSSKYQQAFTPSPGAR (0.3) |  |  |  |  | DCEGDC (0.1) | |
|  |  |  |  |  |  | VGSSKYLQSFTPSPGAR (0.3) |  |  |  |  |  |  |
|  |  |  |  |  |  | VGSSKYQQSFTPNPGAR (0.3) |  |  |  |  |  |  |

North American Avian Lineage Antigenic Site Variants. Variant (Percent prevalence)
