# Supplementary material for: Broadly-Reactive Neutralizing and Non-neutralizing Antibodies Directed against the H7 Influenza Virus Hemagglutinin Reveal Divergent Mechanisms of Protection
Source: PLoS Pathog. 2016 Apr 15;12(4):e1005578. doi: 10.1371/journal.ppat.1005578 (PMC4833315; doi:10.1371/journal.ppat.1005578)
Supplement: S4 Table — (DOCX) [file ppat.1005578.s013.docx]

| Virus | LD_50_ (PFU) | backbone^1^ | pathogenicity decrease over wild type |
| --- | --- | --- | --- |
| Shanghai13 | 10^3.24^ | - | - |
| E65K 1H5/1H10 escape | 10^4.38^ | Shanghai13 | 13.6 fold |
|  |  |  |  |
| Shanghai2 | 10^4.51^ | - | - |
| 1B2 escape | 10^5^ | Shanghai2 | 3.1 fold |
| 1A8 escape | >10^5^ | Shanghai2 | >3.1 fold |

Supplementary table 4: LD_50_ values of wild type viruses and escape mutants in BALB/c mice

^1^This refers to the HA. All viruses were rescued in the backbone of the safe vaccine strain A/PR/8/34.
